# Predicting chemosensitivity using drug perturbed gene dynamics

**DOI:** 10.1186/s12859-020-03947-y

**Published:** 2021-01-07

**Authors:** Joshua D. Mannheimer, Ashok Prasad, Daniel L. Gustafson

**Affiliations:** 1 School of Biomedical Engineering, Colorado State University, Fort Collins, CO USA; 2 Flint Animal Cancer Center, Colorado State University, Fort Collins, CO USA; 3 Department of Chemical and Biological Engineering, Colorado State University, Fort Collins, CO USA; 4 Department of Clinical Sciences, Colorado State University, Fort Collins, CO USA; 5 University of Colorado, Cancer Center Developmental Therapeutics Program, University of Colorado, Aurora, CO USA

**Keywords:** Machine learning, Chemotherapy, Genomics models, Drug response, Cancer, NCI60

## Abstract

**Background:**

One of the current directions of precision medicine is the use of computational methods to aid in the diagnosis, prognosis, and treatment of disease based on data driven approaches. For instance, in oncology, there has been a particular focus on development of algorithms and biomarkers that can be used for pre-clinical and clinical applications. In particular large-scale omics-based models to predict drug sensitivity in in vitro cancer cell line panels have been used to explore the utility and aid in the development of these models as clinical tools. Additionally, a number of web-based interfaces have been constructed for researchers to explore the potential of drug perturbed gene expression as biomarkers including the NCI Transcriptional Pharmacodynamic Workbench. In this paper we explore the influence of drug perturbed gene dynamics of the NCI Transcriptional Pharmacodynamics Workbench in computational models to predict in vitro drug sensitivity for 15 drugs on the NCI60 cell line panel.

**Results:**

This work presents three main findings. First, our models show that gene expression profiles that capture changes in gene expression after 24 h of exposure to a high concentration of drug generates the most accurate predictive models compared to the expression profiles under different dosing conditions. Second, signatures of 100 genes are developed for different gene expression profiles; furthermore, when the gene signatures are applied across gene expression profiles model performance is substantially decreased when gene signatures developed using changes in gene expression are applied to non-drugged gene expression. Lastly, we show that the gene interaction networks developed on these signatures show different network topologies and can be used to inform selection of cancer relevant genes.

**Conclusion:**

Our models suggest that perturbed gene signatures are predictive of drug response, but cannot be applied to predict drug response using unperturbed gene expression. Furthermore, additional drug perturbed gene expression measurements in in vitro cell lines could generate more predictive models; but, more importantly be used in conjunction with computational methods to discover important drug disease relationships.

## Background

A major focus of cancer treatment is the utilization of phenotypic characteristics that can inform data-driven treatment protocols to target specific vulnerabilities of a patient’s cancer [1]. There has been a substantial amount work to characterize the genomic and mutational landscape of cancer that have resulted in successful interventions in cancers, harboring specific mutations or genomic signatures [2–4]. Nonetheless, for the majority of cancers specific genomic prognostic indicators informing treatment have yet to be discovered with current estimates of only ~ 15% of cancer patients being eligible for genome-informed treatment [5]. Cancer is a complex disease that arises from both numerous and diverse biological interactions. Developments in high-throughput drug screening and genomic profiling have laid a solid foundation for characterizing the pharmacogenomic landscape of the disease [6, 7]. Even so, developing specific experimental protocols in vitro or in vivo that probe the entirety of this landscape is an infeasible if not impossible task. A major goal of computational and systems biology has been to integrate and leverage the information inherent in available data to foster new insight about complex biological systems [8]. Specifically in cancer, statistical, mathematical and computational approaches, are starting to be utilized to uncover complex drug-disease relationships [9, 10]. However, this is an inherently complex task. The genome is innately a high dimensional space, has built in redundancy between genes, and gives rise to several complex multivariate interactions many of which we have little or no knowledge about. Thus, identifying these relationships requires developing tools and approaches for deconvolution and screening of this complex data pool.

Recently, a clinical trial in human bladder cancer was concluded using computational methods to leverage cell line data to predict prognosis for neoadjuvant chemotherapy [11]. The origin of these studies has been driven by similar in silico models predicting drug response for in vitro cell lines [12, 13]. One of the most comprehensive evaluations of these methods was conducted as a team-based competition where 44 teams using a variety of different computational approaches competed to predict drug response for 28 therapeutic agents in a panel of 53 breast cancer cell lines [14]. The study concluded that computational approaches could predict drug response using omics data particularly with a high regard to genomics data.

Pan-cancer models have also been shown to predict the response of cytotoxic chemotherapies in large cell line databases such as Genomics for Drug Sensitivity [15] and the National Cancer Institute 60 cell database (NCI60) [16]. However, one of the central findings in [16] was that the predictive capabilities of these models was largely driven by associations between certain drugs that had stratified drug response based on histotype; drugs for which drug response was mostly independent of histotype tended to perform poorly in the models compared to those that did. The dimensionality inherent in omics data makes the model more susceptible to weaker broader signals making smaller, yet more informative signals, hard to isolate. The processes in a cell are inherently dynamic and adaptive. With respect to cancer drugs, the purpose of a drug is to interfere with the dynamic and adaptive mechanisms that are responsible for disease pathology. Therefore, it is reasonable to assume that changes in gene expression after drug perturbation would, in part, be reflective of the underlying mechanisms responsible for drug response. The idea that changes in gene expression are linked to drug mechanism has been reflected in the connectivity map [17, 18] which has shown to give relevant pharmacogenomic insights [19, 20]. Additionally, there have been studies that have leveraged specific gene dynamics in p53 pathway to predict drug response with promising results [21]. These results suggest that perturbation-based models have the potential to reflect drug response. Furthermore, features identified in perturbation-based models may be predictive even when applied to basal gene expression.

The NCI Transcriptional Pharmacodynamic Workbench [22] is a web based tool that allow users to explore the relationship between changes in gene expression, drug response, and drug exposure for 15 different drugs in the NCI60 panel of cell lines. However, this tool only allows a univariate analysis by correlation of gene expression and drug response. To the best of our knowledge, no one has applied multivariate predictive models using this data. We use Support Vector Regression with a radial basis function (SVR-RBF) to build predictive models of drug response for the data available in the NCI Transcription Pharmacodynamic Workbench. Specifically, there is an emphasis on the predictive capabilities of gene expression under different drug treatments. Additionally, the predictive relationships between these datasets are explored using correlation based feature selection [23]. Finally, network-based analysis is utilized to explore the relationships that exist between selected genes for both basal gene expression and drug induced changes in gene expression.

## Results

### Perturbed gene expression at 24 h is a good predictor of drug response

**Fig. 1:**
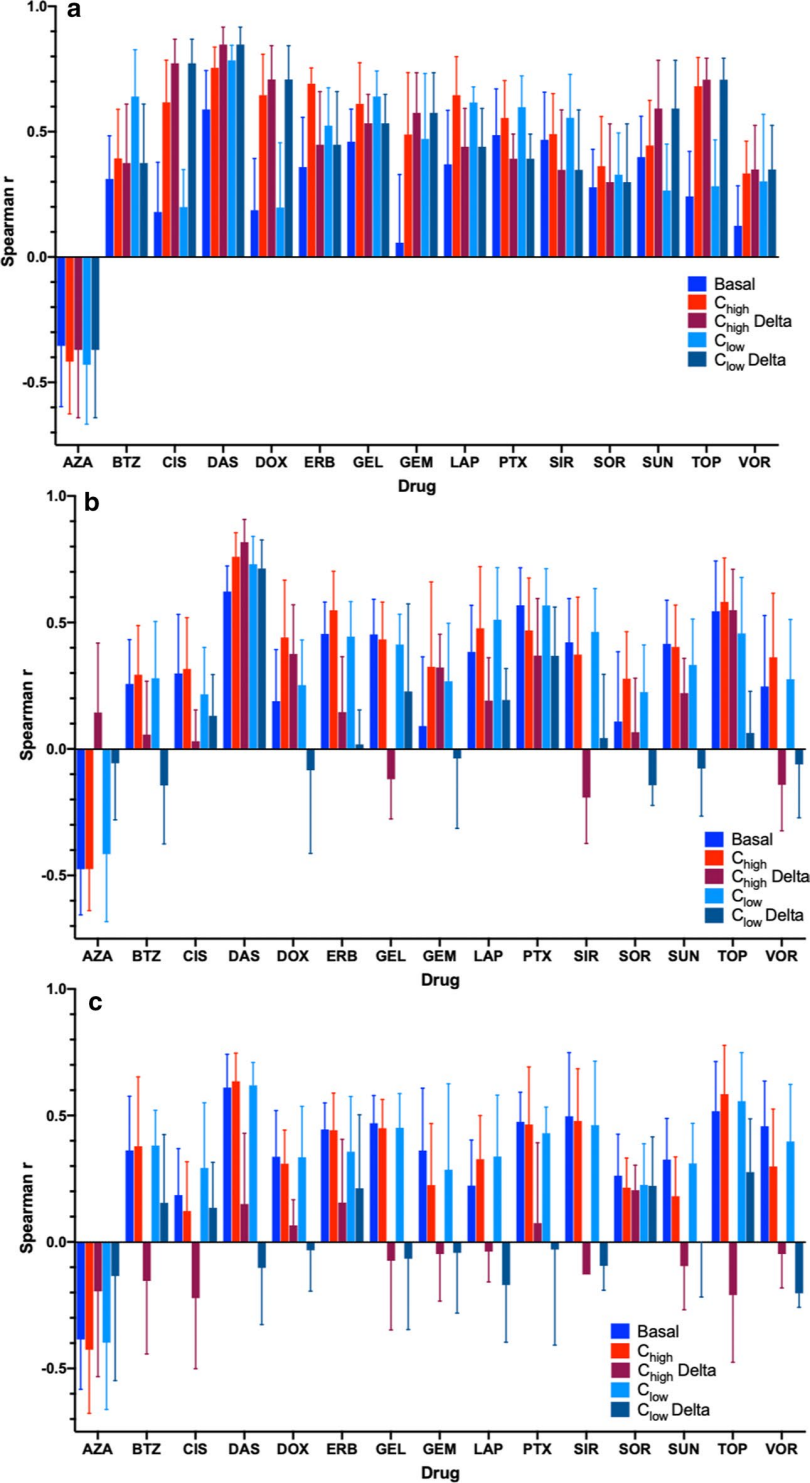
Average Spearman Correlations and with standard of deviation for models using different gene expression profiles for A. 24 h, B. 6 h, and C. 2 h

It can be hypothesized that for each drug there is some timescale for each drug when drug induced perturbation is most predictive of drug response. Using the basal and perturbed gene expression at 2,6, and 24 h, 135 models (9 models for all 15 drugs), per timepoint, were constructed for each gene expression profile (basal, perturbed, expression deltas) for the 3 different treatment conditions. The best performing models, by average spearman correlation, consisted of gene expression profiles from C_high_ gene expression ($\overline{r} =$ 0.495) after 24 h of treatment while, performance was lowest for ΔC_high_ ($\overline{r} =$ 0.025) 2 h post treatment. Performance was dominated by gene expression profiles 24 h post treatment (ΔC_high_, ΔC_low_, C_high_, C_low_) Fig. 1. The highest achieved average spearman correlation was achieved for the drug Dasatinib ($\overline{r} =$ 0.848) using ΔC_high_ gene expression 24 h post treatment compared to lowest for Azacytidine ($\overline{r} =$ 0.144) using ΔC_high_ gene expression 6 h post treatment. The average correlation of the top performing models for each drug drugs was $\overline{r}$ = 0.6074 (SD: 0.16) (Table 1.).

**Table 1:** The dataset that gave the best correlation for each drug

Drug	Abbreviation	Dataset	Correlations
Azacytidine	AZA	C_high_ Delta 6 h	0.144
Bortezomib	BTZ	C_low_ 24 h	0.603
Cisplatin	CIS	C_high_ Delta 24 h	0.773
Dasatinib	DAS	C_high_ Delta 24 h	0.848
Doxorubicin	DOX	C_high_ Delta 24 h	0.709
Erlotinib	ERB	C_high_ 24 h	0.692
Geldanamycin	GEL	C_low_ 24 h	0.641
Gemcitabine	GEM	C_high_ Delta 24 h	0.576
Lapatinib	LAP	C_high_ 24 h	0.646
Paclitaxel	PTX	C_low_ 24 h	0.6
Sirolimus	SIR	C_low_ 24 h	0.556
Sorafenib	SOR	C_low_ Delta 24 h	0.566
Sunitinib	SUN	C_high_ Delta 24 h	0.593
Topotecan	TOP	C_high_ Delta 24 h	0.707
Vorinostat	VOR	0 nM 2 h	0.457

**Fig. 2:**
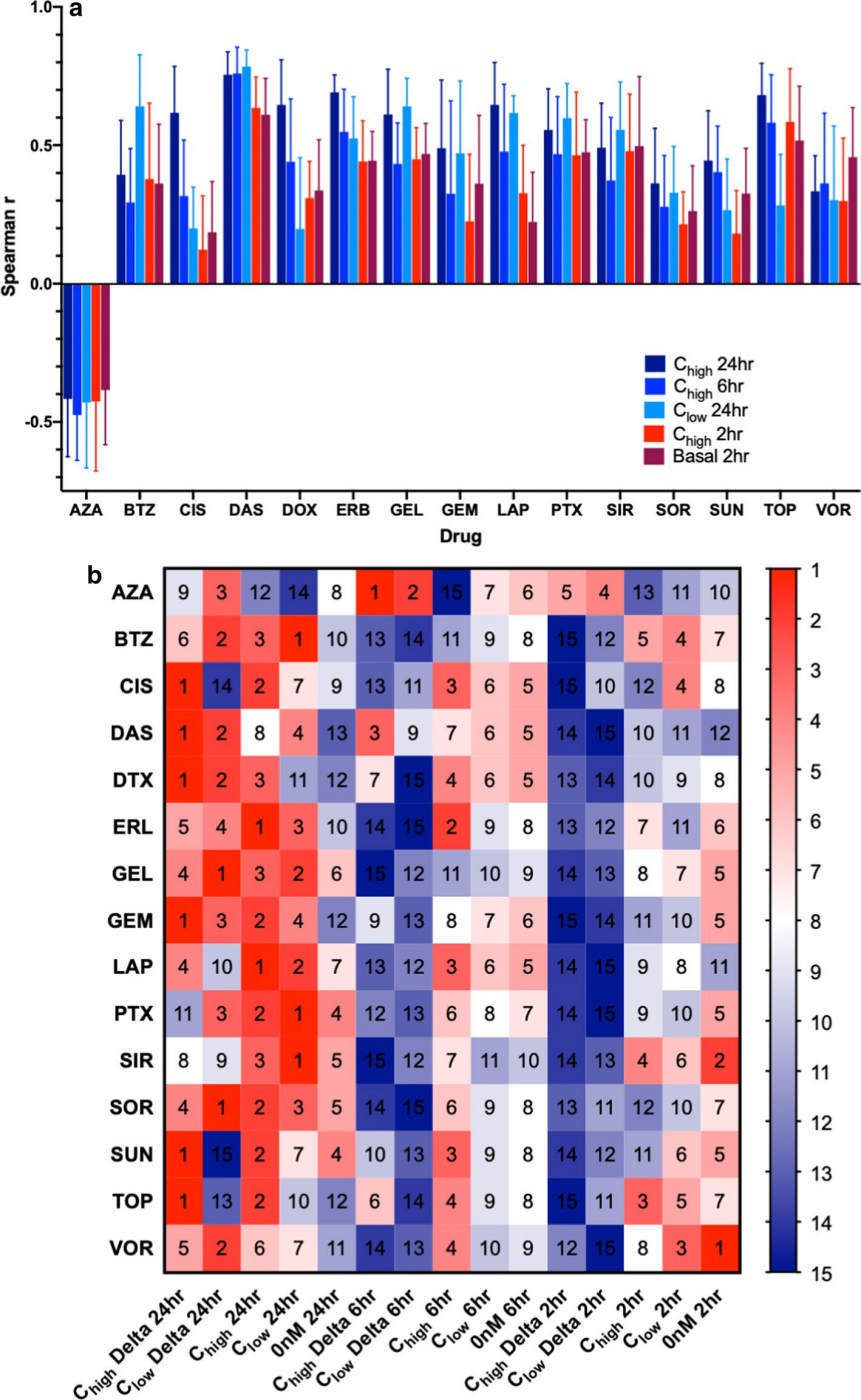
**a** Average spearman correlation plotted such that for each drug exposure is highest on the left and lowest on the right. **b** The rank, in terms of average Spearman correlation, of each model with respect to the gene expression profile used

With respect to each drug and gene expression profile, six drugs were most predictable using ΔC_high_ gene expression at 24 h post treatment, four drugs using C_low_ gene expression at 24 h post treatment, two drugs at using C_high_ gene expression at 24 h post treatment, and a single drug using ΔC_low_ gene expression at 24 h post treatment (Table 1, Fig. 2b). Azacytidine and vorinostat were the only two drugs that did not have the best performance with 24-h post treatment gene expression. Azacytidine was most predictive using ΔC_high_ 6 h post treatment and vorinostat was best predicted by basal gene expression. The interplay between dosage and timing was explored, since some drugs may display more predictive signatures at a low dose and others at a high dose. We found that across all models, gene expression profiles drugged at a high concentration (C_high_/ΔC_high_) performed better than similar gene expression drugged with a lower concentration of drug (C_low_/ΔC_low_). Both C_high_/C_low_ (Δr = 22%) and ΔC_high_/ΔC_low_ (Δr = 0.172%) had large differences in average correlation, however, only C_high_/C_low_ was significantly different (p_wc_ = 0.0005) by Wilcoxen paired t test. Specifically, at 24 h post treatment C_high_ resulted in models 1.2% better then ΔC_high_ gene expression, however the difference was not significant (p_wc_ = 0.427). Conversely, at the lower concentration ΔC_low_ outperformed C_low_ by 2.88% but not significantly (p_wc_ = 0.65). At the 24 h time point models using basal data gene expression performed significantly lower with respect to C_high_ (42.7%, p_wc_ < 1e-4) and ΔC_high_ (42%, p_wc_ < 1e-4). The results were similar at the lower concentration for C_low_ ( 30%, p_wc_ < 1e-4) and ΔC_low_ (32%, p_wc_ < 1e-4). With respect to drug exposure (dose × time), C_low_ at 24 h performs 4.4% better than C_high_ expression at 6 h despite that drug exposure at C_high_ is greater; however this difference is not significant (p_wc_ = 0.1065) (Fig. 2a). Additionally, while noting that C_high_ gene expression at 6 h post treatment performs better than basal data (18%, p_wc_ = 0.0001). C_low_ gene expression at 6 and 2 h and C_high_ gene expression at 2 h post treatment are comparable to models using basal gene expression ranging for 3.4% to 9.8% with no significant difference (p_wc_ > 0.25).

### A smaller set of differentially expressed genes are sufficient to capture drug response

**Fig. 3:**
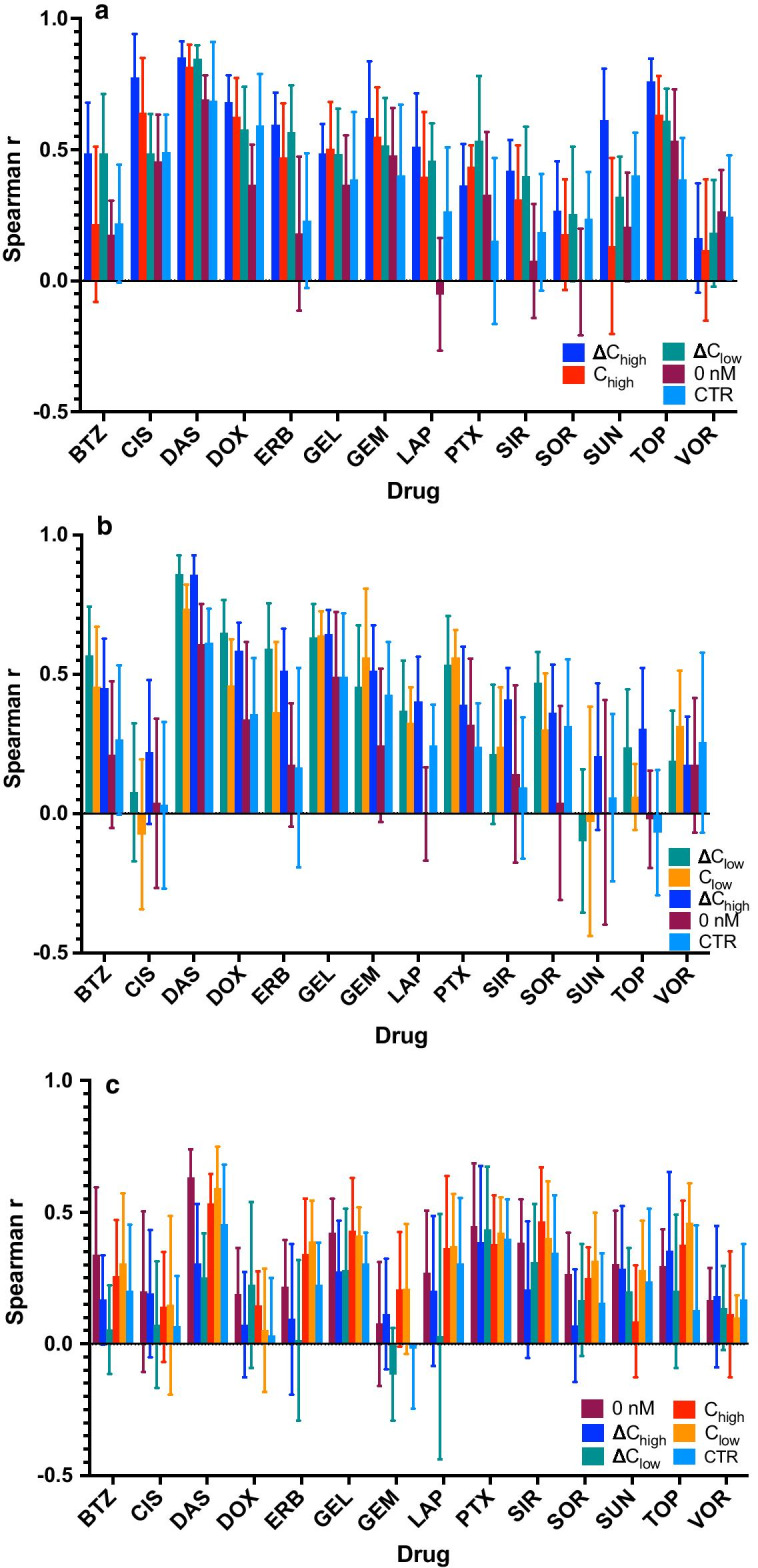
DEGs and gene expression model performance. **a** Models using ∆C_high_ gene expression with features selected from the different (by bar color) gene profiles. **b** Models using ∆C_low_ gene expression with features selected from different gene profiles. **b** Models using 0 nM gene expression with features selected from different gene profiles. Similar plots for C_high_ and C_low_ can be found in Additional file 1: Figure S4

Since each drug has specific modes of action that endow it with cytotoxicity, a smaller set of features, or gene expression signatures, may be as predictive of drug response as the entire ensemble of gene expression. To determine whether a predictive drug response gene expression signature could be found, DEGs were selected for each 24-h gene expression profile and models based on these DEGs were constructed within each gene expression profile (Fig. 3.). DEG gene expression profiles resulted in lower average spearman correlation compared to using all genes (NOFS), with the exception of the ΔC_high_ data (Azacytidine was left out in the analysis as it varied greatly between different testing sets within each individual gene expression profile). The increase in performance while using ΔC_high_ DEGs on ΔC_high_ data compared to the entire ΔC_high_ profile (NOFS) was modest (DEG $\overline{r} =$ 0.5415, NOFS $\overline{r} =$ 0.5294) and not significant (p_wc_ = 0.3437). Additionally, when comparing ΔC_low_ gene expression the performance was only slightly less using DEGs ($\overline{r} =$ 0.4092) then NOFS ($\overline{r} =$ 0.4443) with no significant difference (p_wc_ = 0.2516). However, with respect of C_high_ profiles to C_low_ gene profiles, the performance of the DEG models was significantly less (C_high_ *p* < 1e−4, C_low_ *p* = 0.0181) with differences in performance of 10% (C_low_) to 16.2% (C_high_). The difference between basal DEG models and basal NOFS models data was insignificant (p_wc_ = 0.0533) where the DEG model performed about 10% worse ($\overline{r} =$ 0.299/0.331). Comparisons between models using DEGs and NOFS model on a drug-by-drug basis can be seen in Fig. 3.

### DEGs selected from different gene expression profiles are not universally predictive when applied across gene expression profiles

The advantage of using perturbation data for feature selection is straight forward; if a gene’s expression changes with exposure to drug there is a higher probability that the gene plays a role in the cells response to that drug. Thus, it is not unreasonable to assume that a gene that has a dynamic response to drug exposure is a good feature to use when modeling. However, often times the gene expression data for in vitro cell lines and tumor samples is available without any exposure to drug. Thus, it might be advantageous to use available drug perturbed data from another dataset to select features with a dynamic response, and apply those features in another dataset. However, it unclear whether a signature derived from drugged gene expression data also reflects drug response under unperturbed conditions. Therefore, one of the essential questions that we explored was the performance of gene signatures derived from one dataset while being applied to gene expression under different drug induced dynamics. In order to explore this question, we used correlation-based feature selection to select features using one gene expression profile and then applied those selected genes in models utilizing a different gene expression profile (refer to methods).

Differentially expressed genes selected in basal gene expression and applied to ΔC_high_ gene expression resulted in a 46.5% drop in performance ($\overline{r} =$ 0.542 to 0.29). For comparison, using 100 randomly selected genes resulted in a smaller drop in performance by only 36%. The difference in performance was minimal (12%) when ΔC_low_ DEGs were applied to ΔC_high_ expression data ($\overline{r} =$ 0.542 to 0.48) and was substantially lower when C_high_ DEGs were applied to the same data, resulting in a drop of 22% ($\overline{r} =$ 0.542 to 0.429) (Fig. 3a). Likewise, with respect to ΔC_low_ data using basal DEGs resulted in a 52% decrease in overall performance ($\overline{r} =$ 0.41 to 0.197); however, contrary to the results for ΔC_high_, when DEGs from ΔC_high_ were applied to ΔC_low_ gene expression performance increased by 5% ($\overline{r} =$ 0.41 to 0.43) but was not significant (p_wc_ = 0.96). The application of C_low_ DEGs to ΔC_low_ gene expression resulted in 16.7% drop ($\overline{r} =$ 0.41 to 0.351,* p* = 0.0028) (Fig. 3b).

We also tested the predictive capability of DEGs selected from basal and expression deltas on perturbation gene expression. Similar to what we found for expression deltas, basal DEGs resulted in the greatest drop in performance for both C_high_ 16.7% ($\overline{r} =$ 0.476 to 0.396) and C_low_ 15.1% ($\overline{r} =$ 0.4253 to 0.425) (Additional file 1: Fig. S4). Performance of ΔC_high_ DEGs on C_high_ gene expression resulted in a slight increase in performance by roughly 1.7% ($\overline{r} =$ 0.476 to 0.484), while ΔC_low_ DEGs had only a negligible effect when applied to C_low_ gene expression ($\overline{r} =$ 0.4253 to 0.4250) (Additional file 1: Fig. S4). The application of C_low_ DEGs to C_high_ gene expression resulted in a decrease of roughly 1.7% ($\overline{r} =$ 0.476 to 0.468), a slightly larger, but still models, drop in performance resulted from the use C_high_ DEGs to C_low_ gene expression ($\overline{r} =$ 0.4253 to 0.414).

DEGs were selected from perturbed gene expression and expression deltas and these DEGs were applied to basal gene expression (Fig. 3c). When ΔC_high_ DEGs were applied to basal genes expression, performance substantially decreased by 30% ($\overline{r} =$ 0.299 to 0.208) and similarly, using ΔC_low_ DEGs on basal gene expression the performance decreased by 44% ($\overline{r} =$ 0.299 to 0.166). Finally, a random selection gene expression from 100 random genes resulted in a decreased performance by only 29% ($\overline{r} =$ 0.299 to 0.213). Furthermore, models using basal gene expression increased performance by 6% ($\overline{r} =$ 0.299 to 0.317) when using DEGs from C_low_ and ΔC_high_ DEGS applied to basal gene expression decreased performance by 2.7%. These results indicate that changes in gene expression that predict drug response are not predictive features in basal gene expression.

### DEGS and network topology

**Fig. 4:**
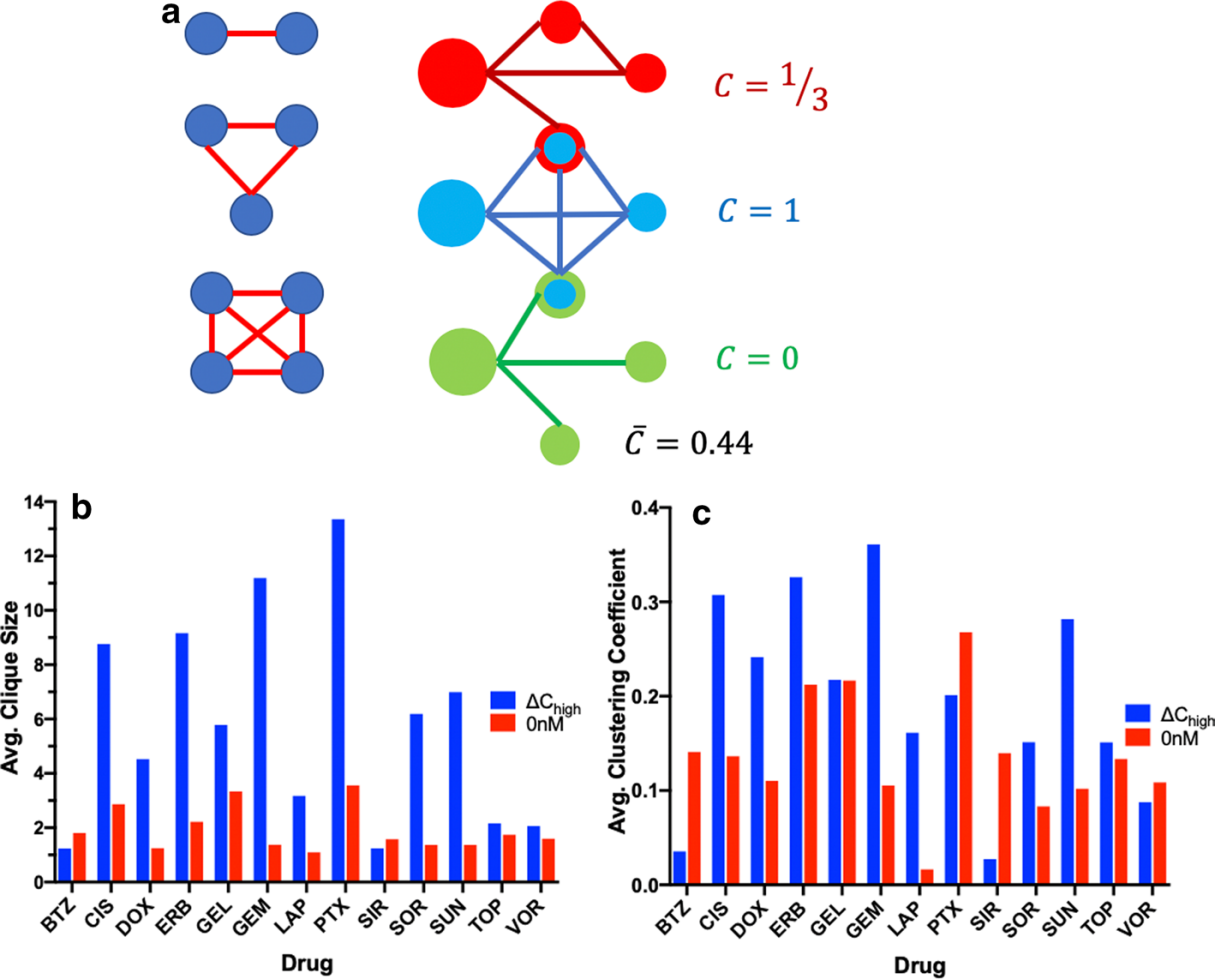
**a** Illustration of a clique (Right) of size 2 (top), 3 (Middle), 4 (bottom) and Clustering Coefficient for 3 different subgraphs along with the average over all three subgraphs. **b** Average clique size, and **c** average clustering coefficient for different drugs for ∆C_high_ and 0 nM at 24 h

One of the fundamental concepts in biology is that cellular systems are an assembly of dynamic interactions forming a network of interacting components, that provide the framework for all functions of the cell. While generally it is well understood that networks encompass some kind of connectivity, network models allow for rigorous mathematical approach to understand and analyze this general notion of connectivity. Particularly, there has been an interest in applying concepts behind network topology to understand the relationship between genes, disease states, and treatments [21, 24]. To explore the relationships between genes under both a basal and perturbed states, DEG networks were constructed using correlations to link genes. As outlined in methods, this was accomplished by calculating a correlation matrix for both the basal and expression delta gene expression profiles. Then Boolean graphs was constructed by placing edges between genes that had a spearman correlation p-value below a Bonferroni corrected significance level. The topology of the given network was then quantified based on cliques, a subset of nodes which share an edge with every other node in the subset, and the clustering coefficient which measures the connectivity of a subset of nodes all sharing an edge with a single node (Fig. 4a).

**Fig. 5:**
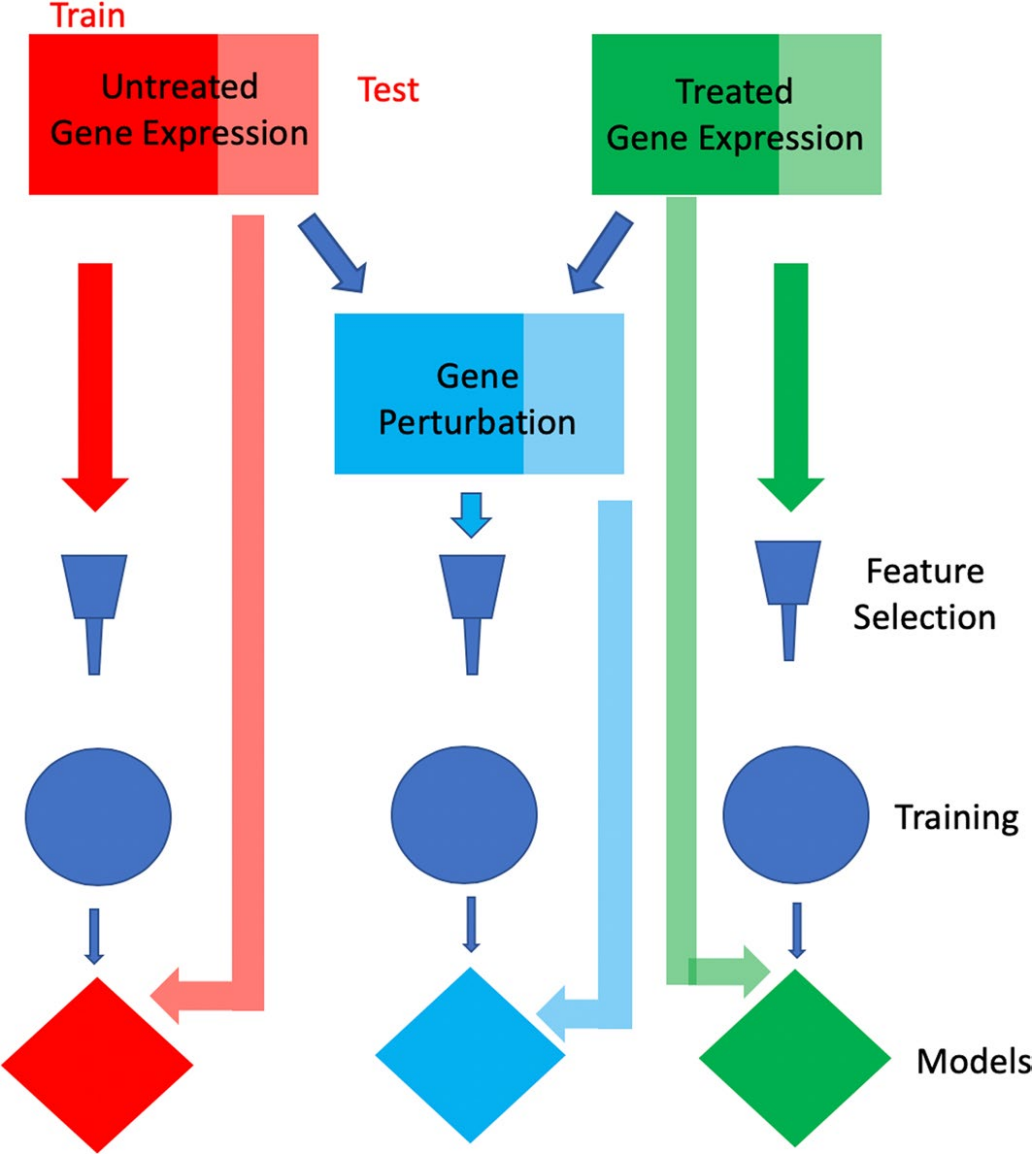
Model building outline

There was a clear distinction between topological properties of networks formed with expression deltas DEGs compared with basal DEGs. On average networks constructed from expression DEGs formed 389% more cliques then networks formed with basal DEGs. The number of cliques which exceeded a size of 2 was also much greater using the expression deltas DEGs averaging around 60% similarly compared to 21% for basal DEGs. Likewise, the average clique length was 314% greater using expression deltas DEGs compared to Basal DEGs (Fig. 5b). Additionally, clique participation was much greater in expression delta. gene networks with each node participating in 1.1% of all cliques compared to only 0.3% for basal DEG networks. Lastly, expression deltas DEG networks had an average clustering coefficient that was 2.15 × greater than that of the basal DEG networks (Fig. 4c). Based on network topological features expression deltas DEGs showed a much greater level of interaction compared to DEGs derived from basal gene expression.

### Clique participation is a signature of cancer and drug response association of genes

In order to explore how clique participation might be associated with cancer association we looked at the 15 genes that participated in the most cliques in both the expression deltas network and the basal gene network. Analysis of expression deltas-based data yielded several genes that had been cited in the literature to have some known association with cancer. For example, bortezomib included the Breast Cancer Metastasis Suppressor 1 gene (BMRS1), which has shown to play a role in metastatic potential in breast [25], melanoma [26], and non-small cell lung cancer [27–30]. Likewise, genes for paclitaxel included RNA Binding Protein 5 (RBM5), active in tumor suppression in breast [33] and lung [35] cancers and has also been associated with p53 activity [34]. The genes of doxorubicin included the gene MOB Family Member 4 (MOB4) which has been associated with tumor progression in glioblastoma [31]. Additionally, genes associated with tumor progression included PBX Homeobox Interacting Protein (PBXIB1), shown to play a role in lung adenocarcinoma [32, 33] and astrocytoma [38] for geldanamycin. Eukaryotic translation initiation factor 4E (EIF4E) was a highly connected gene for Doxorubicin, and has been shown to promote tumor progression upon phosphorylation in breast cancer and lymphoma cells [34–36]. Apoptosis related genes including Nuclear Receptor Coactivator (NCOA1) in cisplatin [37] and TIMELESS interacting protein (TIPIN), for lapatinib [38, 39]. Additionally, Cell Division Cycle 25A (CDC25A), a gene involved in cell cycle regulation in cancer [40] was a DEG for geldanamycin. Likewise, Topoisomerase DNA binding Protein (TOPBP1), a highly connected paclitaxel DEG, has been shown to play a regulating role in G2-M phase cell cycle progression [41]. Furthermore, erlotinib included the gene survivin (BIRC5) and geldanamycin included RAN binding protein (RANBP1) both of which have been associated with paclitaxel sensitivity [42, 43]. These represent a subset of genes that we identified. For all drugs, genes with cancer associated references could be found or genes involved in cell cycle regulation, apoptosis, or translation. A list of these additional genes can be found in the supplementary material.

Likewise, several genes with maximum clique participation in the basal gene networks had associations with cancer. The gene that participated in the most cliques for bortezomib was ABCE1, a known mediator of drug resistance [44–46]. Likewise, for lapatinib ATB binding cassette family F member 1 (ABCF1) also associated with chemoresistance [47]. One of the genes picked up for sorafenib was EGFR and among the genes for topotecan was a tumor suppressing genes ST14 (matriptase) [48–50]. Additionally, R-Ras 2, an oncogene gene known to be associated with tumorigenesis and metastasis was present for erlotinib [51, 52]. Additional genes of interest from basal gene expression networks can be found in the supplementary material.

## Discussion

Small molecule gene perturbation has become a new focus to understand the relationships between diseases and drugs [17, 18, 22]. One of the central roles of this work was to understand how gene dynamics could inform drug response and what roles drug exposure may play. The results suggest, given a limited subset of drugs and cell lines, that the differences in gene expression at 24 h between untreated and treated cells with a relatively higher high dose of drug is the best predictor of drug response compared to basal gene expression or perturbed responses at earlier time points and lower drug doses. Additionally, the data suggests that elapsed time might play may be a bigger factor than exposure, as models that utilized perturbation gene profiles treated with a low dose at the 24-h post treatment often outperformed models using high dosed gene expression but at an earlier time point. The similarity between the predictive ability of non-drugged gene expression and drugged gene expression at 2 and 6 h suggest that changes in gene expression are rather minimal at these early time points. One of the questions that might be of interest would be to answer at exactly what time do changes in gene expression become predictive and whether time points greater than 24 h could possibly be more predictive? Additionally, if the changes in gene expression could be measured with enough temporal resolution it might enable analysis of gene trajectories to see if they are indicative of drug response. Additionally, there is an interesting question that arises, are mechanisms of drug response dose dependent? For example, some cancers might have a certain tolerance to drug concentration and only after that concentration has been exceeded is there a coordinated response in gene expression. This might explain why gene expression is more predictive at higher doses than lower doses, higher doses initiate different genomic responses. However, there is only a minimal performance increase in our models, suggesting that this kind of effect, at least at the drug doses explored, has minimal effect. Nonetheless, future work may include a more thorough analysis of changes in gene expression with respect to levels of drug exposure.

The role of feature selection in omics-based models is somewhat controversial. In certain other data-driven models feature selection can be critical in eliminating noise, resulting in a better performing model. In genomics two of the most used methods of feature selection is to use user-defined genes, for example, exploiting all genes known to be in a specific pathway or leveraging statistical inference to select features that can most likely explain the variability in the phenomena, such is the case with CBF or a pairwise t-test like LIMMA [53]. With respect to the former, this method inherently gives biological context; however, the problem of predicting drug response is that many of the mechanisms of drug response are not known [54], thus making it difficult to select genes based on prior knowledge. The second approach is very susceptible to noise and it is not clear if there is any advantage over using all features [14, 16]. However, the use of all features lacks the specificity to be useful for hypothesis generation. The inferential approach is somewhat of a go between, it does not exclude genes based on any prior bias, but preferences features only by the statistical capability to explain variation with respect to some biological observation such as drug response. The hope is that many of these features have a shared underlying biological context which results in the observed statistics. Drug response has both static and dynamic components, for example, multi drug resistance can result from the overexpression of efflux transporters [54] and down regulation of deoxycytidine kinase is seen in gemcitabine resistance [55]. A central question that underlies the DEG experiments is whether statistical inference can capture an underlying relationship between static and dynamic gene expression when it comes to drug response. Based on our results, the drug induced changes in gene expression that best predicted drug response did not have any predictive power in basal gene expression and the same was true of basal gene expression with respect to gene changes. In some instances, this might be indicative of how some signaling networks are designed. For example, if the protein expressed by gene A regulates the expression of gene B and a drug targets the enzymatic function of protein A, the expression of gene A, barring a feedback mechanism, would not change. Therefore, the downstream effects of the drug are exhibited by the change in expression of gene B. Therefore, the basal expression of gene B would not be indicative of drug response and there would be no dynamic response of gene A. However, this is only in respect to univariate feature selection, it is perfectly reasonable to believe that features exist in a higher dimensional space which might pertain to an underlying biological phenomenon; however, methods to find such features and map them to specific genes are yet to be developed.

The complexities of cancer therapy are immense and thus, especially from a pharmacological view, it is necessary to understand what properties make a cancer susceptible to a certain drug or what mechanisms are responsible for resistance. A recent publication showed that cell proliferation could be maintained without the specific proteins targeted by many therapies; additionally, they also demonstrated that many of these drugs achieved cytotoxicity without the inclusion of the druggable target [56]. Targeted therapies are notorious for an initial response followed quickly by developed resistance [57, 58]. Furthermore, models using only target data performed substantially worse in both gene expression profiles (Additional File 1). All in all, gene expression of drug targets in both perturbed and unperturbed data are poor predictors of drug response. However, through network analysis we found that genes with high clique participation had been associated with cancer in both changes in gene expression and basal gene expression. Furthermore, the networks constructed from changes in gene proved to be significantly more connected networks than similar networks constructed from basal gene expression. This might explain why perturbation is a better predictor of drug response, the redundancies that result from coordinated changes lead to a stronger signal to noise ratio. Alternatively, this might suggest that drug response is a more likely a function of several interacting genes and several different mechanisms and this is reflected better under dynamic changes. This is certainly consistent with the observation that cancer has multiple mechanisms of drug resistance [59, 60].

## Conclusion

There are several roles for computational models in oncology including, but not limited to, patient prognostics and treatment, new treatment development, informing clinical trials, and as a method of hypothesis generation especially when it comes interaction between cellular processes and drug mechanisms. Genetic perturbations would be difficult to leverage in a clinical setting, a prognostic model to aid patient treatment needs to be quick and cost effective. Acquiring, gene perturbations of a patient’s tumor for multiple drugs would be both difficult and time consuming. In vitro drug screens are often the first step in determining the potential of a possible drug candidate and also serve as a platform for hypothesis generation. However, as our data indicate, obtaining predictive models with basal gene expression is difficult and furthermore might not be the best data to determine drug mechanism. However, gene perturbations prove to be better at capturing drug response and they exhibit a high level of connectivity between genomic features. Therefore, modeling in vitro gene expression changes could be instrumental to better understand the dynamic mechanisms behind drug response. A better understanding of the underlying gene dynamics induced by a drug would most likely promote insight into the underlying genetic mechanisms of drug response which are essential for developing new treatments and building robust prognostic models at the clinical level. In order to fully take advantage of this strategy it would be essential to generate more perturbation data similar in scope to the genomic and drug profiling that is associated with large cell line panels such as the GDSC, CCLE, and expanded among more drugs in the NCI60.

## Methods

### Data acquisition and pre-processing

The Affymetrix U133A 2.0 raw expression data from Monks et.al was downloaded from the gene expression omnibus (https://www.ncbi.nih.gov/geo) series number GSE116436 [22]. Each drug had CEL files for gene expression for untreated cell lines (basal, 0 nM), cell lines drugged at a low dose of drug (C_low_), and cell lines treated with a high dose of drug (C_high_) at 2,6, and 24 h. Frozen robust multi-array analysis (fRMA)[61] was performed using the fRMA bioconductor (version 3.8.0) package in R (version 3.5.1) for all CEL files corresponding to each individual drug. At 2,6, and 24 h the data was split into gene expression matrices for basal, C_low_, and C_high_. All gene expression matrices were scaled to a mean of 0 and unit standard deviation. Perturbation gene expression was obtained by subtracting the basal data from the drug treated data (C_high_,C_low_) yielding matrices of gene differences at the high and low concentration (ΔC_high_, ΔC_low_). Throughout the text (C_high_, C_low_) as perturbation gene expression and (ΔC_high_, ΔC_low_) as perturbed gene expression deltas or simply expression deltas. NCI60 drug response data was obtained from the CellMiner version 2.2 https://discover.nci.nih.gov/cellminer [62]. The natural log of the GI50 was averaged for all measurements attributed to the same single cell line giving a single average LN GI50 for each cell line per drug.

Training and validation sets were generated randomly using threefold nested cross validation. In order to generate a robust measure of performance across all gene expression datasets this process was repeated 2 more time giving a total of 9 random training and validation pairs with each cell line being represented at exactly 3 times during validation. Thus, amounting to 9 replicates for each gene expression matrix (basal/0 nM, C_low_, C_high_, ΔC_low_, ΔC_high_) (Fig. 5) at 2, 6, and 24 h.

### Modeling

All models were trained using ε-insensitive Support Vector Regression (SVR) using a radial basis kernel function (RBF) from Scikit-learn [63] version 20.3 in python version 3.7.3.

Parameters were optimized using a tenfold random shuffle cross validation scheme on subsets of the training set. Differentially expressed genes (DEGs) were chosen using correlation based feature selection (CBF) [7] using spearman correlation in scipy version 1.2.1. For the DEGs models, the DEGs chosen are the identity of the genes, however, the gene expression data used in the model will be from a possibly different dataset. For example, if the DEGs are from the C_high_ data set, but the model is being evaluated on ΔC_low_ the gene expression, the model uses gene expression from ΔC_low_ but only genes that selected from the C_high_ are used. The performance of each model is quantified using spearman correlation between the predicted and measured values. The general performance, as displayed in the manuscript figures, is given as the average spearman correlation and standard of deviation of the models over all 9 test sets for a given drug. This approach allows for a point and range estimate given a random sample of cancer cells from a larger population of possible cancer cells. The spearman correlation for each individual model can be sound in additional files 2 though 6 Graphics are generated using Prism Version 8 and to calculate significance, the paired Wilcoxen t test is used in comparing different models.

### Topological network analysis

A graph, a mathematical formalism that represents networks, is defined as an ordered set of nodes, *V*, and the edges, *E*, that connect nodes $G\left( {V,E} \right)$. The frequency and orientation with which two nodes are connected describe the networks topology. A topological measurement, cliques, are a subset of nodes such that every node in the subset shares an edge with every other node in the subset (Fig. 4a). Additionally, a graphs topology can be described by a connectivity coefficient which is a measurement of the degree to which a node is connected to other nodes (Fig. 4a). We use these graph theoretic principles to estimate networks of genes. Graphs, gene networks, are constructed as follows; using the smallest DEGs from all nine training/validation sets an adjacency matrix was obtained by first constructing a correlation matrix using spearman r. An edge was considered to connect two nodes if the p-value for the spearman correlation met the Bonferroni corrected cutoff *p* value (α < 0.05). Using this criteria an adjacency matrix can be constructed where each entry in the matrix has a one, if genes i and j meet the criteria for an edge between genes i and j, and a zero otherwise The software NetworkX 2.4 [64] was used to generate a undirected graph from the adjacency matrix and calculate to cliques and average clustering coefficients of the generated graphs.

## Supplementary information

**Additional file 1:** Supplementary Materials.

**Additional file 2:** Raw Data for Figures 1 and 2.

**Additional file 3:** Additional Data for Figures 1 and 2.

**Additional file 4:** Spearman correlations featured in Figure 3.

**Additional file 5:** Figure 3 Spearman p-values.

**Additional file 6:** Maximum absolute differences (MAD) for Figure 3.

**Additional file 7:** Gene Deltas Network Analysis presented in Figure 4.

**Additional file 8:** Basal Gene Network Analysis presented in Figure 4.

## Data Availability

All summary data included in results is included in supplementary material. All data generated in this study are available upon request, and have not been included because of the sheer volume of data generated, but will be readily available if requested. The gene expression data used in the models can be obtained the gene expression omnibus (https://www.ncbi.nih.gov/geo) series number GSE116436. The drug response data can be obtained from the CellMiner version 2.2 https://discover.nci.nih.gov/cellminer. Python 3.7.3 and SciKit-learn version 20.3 are both included in the Anaconda platform which can be downloaded at https://docs.anaconda.com/anaconda/install/. The software NetworkX is available at https://networkx.github.io/documentation/latest/.

## Abbreviations

CBF: Correlation based feature selection
CCLE: Cancer cell line encyclopedia
C_low_: Gene expression after the lowest concentration of drug was applied to cells
C_high_: Gene expression after the lowest concentration of drug was applied to cells
∆C_low_: Basal gene expression subtracted from C_low_ gene expression
∆C_high_: Basal gene expression subtracted from C_high_ gene expression
DEG: Differentially expressed gene
GDSC: Genomics for drug sensitivity in cancer
NCI60: National Cancer Institute 60 cell line panel
RBF: Radial basis kernel function
SVR: Support Vector Regression

## References

[1] Collins FS, Varmus H (2015). A new initiative on precision medicine. N Engl J Med.

[2] Cutter GR, Liu Y (2012). Personalized medicine: the return of the house call?. Neurol Clin Pract.

[3] Toi M, Iwata H, Yamanaka T, Masuda N, Ohno S, Nakamura S, Nakayama T, Kashiwaba M, Kamigaki S, Kuroi K (2010). Clinical significance of the 21-gene signature (Oncotype DX) in hormone receptor-positive early stage primary breast cancer in the Japanese population. Cancer.

[4] Romond EH, Perez EA, Bryant J, Suman VJ, Geyer CE, Davidson NE, Tan-Chiu E, Martino S, Paik S, Kaufman PA (2005). Trastuzumab plus adjuvant chemotherapy for operable HER2-positive breast cancer. N Engl J Med.

[5] Marquart J, Chen EY, Prasad V (2018). Estimation of the percentage of US patients with cancer who benefit from genome-driven oncology. JAMA Oncol.

[6] Scherf U, Ross DT, Waltham M, Smith LH, Lee JK, Tanabe L, Kohn KW, Reinhold WC, Myers TG, Andrews DT (2000). A gene expression database for the molecular pharmacology of cancer. Nat Genet.

[7] Schena M (1996). Genome analysis with gene expression microarrays. BioEssays.

[8] Werner HMJ, Mills GB, Ram PT (2014). Cancer systems biology: a peek into the future of patient care?. Nat Rev Clin Oncol.

[9] Fessele KL (2018). The rise of big data in oncology. Semin Oncol Nurs.

[10] Altrock PM, Liu LL, Michor F (2015). The mathematics of cancer: integrating quantitative models. Nat Rev Cancer.

[11] Flaig TW, Tangen CM, Daneshmand S, Alva AS, Lerner SP, Lucia MS, McConkey DJ, Theodorescu D, Goldkorn A, Milowsky MI et al. SWOG S1314: A randomized phase II study of co-expression extrapolation (COXEN) with neoadjuvant chemotherapy for localized, muscle-invasive bladder cancer. J Clin Oncol. 2019, 37(15_suppl):4506–4506.

[12] Garnett MJ, Edelman EJ, Heidorn SJ, Greenman CD, Dastur A, Lau KW, Greninger P, Thompson IR, Luo X, Soares J (2012). Systematic identification of genomic markers of drug sensitivity in cancer cells. Nature.

[13] Barretina J, Caponigro G, Stransky N, Venkatesan K, Margolin AA, Kim S, Wilson CJ, Lehár J, Kryukov GV, Sonkin D (2012). The Cancer Cell Line Encyclopedia enables predictive modelling of anticancer drug sensitivity. Nature.

[14] Costello JC, Heiser LM, Georgii E, Gönen M, Menden MP, Wang NJ, Bansal M, Ammad-ud-din M, Hintsanen P, Khan SA (2014). A community effort to assess and improve drug sensitivity prediction algorithms. Nat Biotechnol.

[15] Yang W, Soares J, Greninger P, Edelman EJ, Lightfoot H, Forbes S, Bindal N, Beare D, Smith JA, Thompson IR (2013). Genomics of Drug Sensitivity in Cancer (GDSC): a resource for therapeutic biomarker discovery in cancer cells. Nucleic Acids Res.

[16] Mannheimer JD, Duval DL, Prasad A, Gustafson DL (2019). A systematic analysis of genomics-based modeling approaches for prediction of drug response to cytotoxic chemotherapies. BMC Med Genomics.

[17] Lamb J, Crawford ED, Peck D, Modell JW, Blat IC, Wrobel MJ, Lerner J, Brunet J-P, Subramanian A, Ross KN (2006). The connectivity map: using gene-expression signatures to connect small molecules, genes, and disease. Science.

[18] Subramanian A, Narayan R, Corsello SM, Peck DD, Natoli TE, Lu X, Gould J, Davis JF, Tubelli AA, Asiedu JK (2017). A next generation connectivity map: L1000 platform and the first 1,000,000 profiles. Cell.

[19] Rho SB, Kim B-R, Kang S (2011). A gene signature-based approach identifies thioridazine as an inhibitor of phosphatidylinositol-3′-kinase (PI3K)/AKT pathway in ovarian cancer cells. Gynecol Oncol.

[20] Sanda T, Li X, Gutierrez A, Ahn Y, Neuberg DS, O'Neil J, Strack PR, Winter CG, Winter SS, Larson RS (2010). Interconnecting molecular pathways in the pathogenesis and drug sensitivity of T-cell acute lymphoblastic leukemia. Blood.

[21] Choi M, Shi J, Zhu Y, Yang R, Cho K-H (2017). Network dynamics-based cancer panel stratification for systemic prediction of anticancer drug response. Nat Commun.

[22] Monks A, Zhao Y, Hose C, Hamed H, Krushkal J, Fang J, Sonkin D, Palmisano A, Polley EC, Fogli LK (2018). The NCI transcriptional pharmacodynamics workbench: a tool to examine dynamic expression profiling of therapeutic response in the NCI-60 cell line panel. Can Res.

[23] Hall M. Correlation-based feature selection for machine learning. New Zealand Waikato University; 1999.

[24] Hopkins AL (2008). Network pharmacology: the next paradigm in drug discovery. Nat Chem Biol.

[25] Seraj MJ, Samant RS, Verderame MF, Welch DR (2000). Functional evidence for a novel human breast carcinoma metastasis suppressor, BRMS1, encoded at chromosome 11q13. Cancer Res.

[26] Riker AI, Samant RS (2012). Location, location, location: the BRMS1 protein and melanoma progression. BMC Med.

[27] Smith PW, Liu Y, Siefert SA, Moskaluk CA, Petroni GR, Jones DR (2009). Breast cancer metastasis suppressor 1 (BRMS1) suppresses metastasis and correlates with improved patient survival in non-small cell lung cancer. Cancer Lett.

[28] Kim B, Nam HJ, Pyo KE, Jang MJ, Kim IS, Kim D, Boo K, Lee SH, Yoon JB, Baek SH (2011). Breast cancer metastasis suppressor 1 (BRMS1) is destabilized by the Cul3-SPOP E3 ubiquitin ligase complex. Biochem Biophys Res Commun.

[29] Zhao XL, Wang P: [Expression of SATB1 and BRMS1 in ovarian serous adenocarcinoma and its relationship with clinieopathological features]. Sichuan Da Xue Xue Bao Yi Xue Ban 2011, 42(1):82–85. 21355308

[30] Rivera J, Megias D, Bravo J (2007). Proteomics-based strategy to delineate the molecular mechanisms of the metastasis suppressor gene BRMS1. J Proteome Res.

[31] Tang F, Zhang L, Xue G, Hynx D, Wang Y, Cron PD, Hundsrucker C, Hergovich A, Frank S, Hemmings BA (2014). hMOB3 modulates MST1 apoptotic signaling and supports tumor growth in glioblastoma multiforme. Cancer Res.

[32] Liu L, Huang J, Wang K, Li L, Li Y, Yuan J, Wei S (2015). Identification of hallmarks of lung adenocarcinoma prognosis using whole genome sequencing. Oncotarget.

[33] van Vuurden DG, Aronica E, Hulleman E, Wedekind LE, Biesmans D, Malekzadeh A, Bugiani M, Geerts D, Noske DP, Vandertop WP (2014). Pre-B-cell leukemia homeobox interacting protein 1 is overexpressed in astrocytoma and promotes tumor cell growth and migration. Neuro Oncol.

[34] Wheater MJ, Johnson PW, Blaydes JP (2010). The role of MNK proteins and eIF4E phosphorylation in breast cancer cell proliferation and survival. Cancer Biol Ther.

[35] Muta D, Makino K, Nakamura H, Yano S, Kudo M, Kuratsu J (2011). Inhibition of eIF4E phosphorylation reduces cell growth and proliferation in primary central nervous system lymphoma cells. J Neurooncol.

[36] Zhou ZJ, Dai Z, Zhou SL, Hu ZQ, Chen Q, Zhao YM, Shi YH, Gao Q, Wu WZ, Qiu SJ (2014). HNRNPAB induces epithelial-mesenchymal transition and promotes metastasis of hepatocellular carcinoma by transcriptionally activating SNAIL. Cancer Res.

[37] Wang L, Yu Y, Chow DC, Yan F, Hsu CC, Stossi F, Mancini MA, Palzkill T, Liao L, Zhou S (2015). Characterization of a steroid receptor coactivator small molecule stimulator that overstimulates cancer cells and leads to cell stress and death. Cancer Cell.

[38] Baldeyron C, Brisson A, Tesson B, Némati F, Koundrioukoff S, Saliba E, De Koning L, Martel E, Ye M, Rigaill G (2015). TIPIN depletion leads to apoptosis in breast cancer cells. Mol Oncol.

[39] Lee JA, Park JE, Lee DH, Park SG, Myung PK, Park BC, Cho S (2008). G1 to S phase transition protein 1 induces apoptosis signal-regulating kinase 1 activation by dissociating 14-3-3 from ASK1. Oncogene.

[40] Li N, Zhong X, Lin X, Guo J, Zou L, Tanyi JL, Shao Z, Liang S, Wang L-P, Hwang W-T (2012). Lin-28 homologue A (LIN28A) promotes cell cycle progression via regulation of cyclin-dependent kinase 2 (CDK2), cyclin D1 (CCND1), and cell division cycle 25 homolog A (CDC25A) expression in cancer. J Biol Chem.

[41] Yamane K, Chen J, Kinsella TJ (2003). Both DNA topoisomerase II-binding protein 1 and BRCA1 regulate the G2-M cell cycle checkpoint. Cancer Res.

[42] Amato R, Scumaci D, D'Antona L, Iuliano R, Menniti M, Di Sanzo M, Faniello MC, Colao E, Malatesta P, Zingone A (2013). Sgk1 enhances RANBP1 transcript levels and decreases taxol sensitivity in RKO colon carcinoma cells. Oncogene.

[43] Zaffaroni N, Pennati M, Colella G, Perego P, Supino R, Gatti L, Pilotti S, Zunino F, Daidone MG (2002). Expression of the anti-apoptotic gene survivin correlates with taxol resistance in human ovarian cancer. Cell Mol Life Sci.

[44] Kara G, Tuncer S, Türk M, Denkbaş EB (2015). Downregulation of ABCE1 via siRNA affects the sensitivity of A549 cells against chemotherapeutic agents. Med Oncol.

[45] Wang L, Zhang M, Liu D-X (2014). Knock-down of ABCE1 gene induces G1/S arrest in human oral cancer cells. Int J Clin Exp Pathol.

[46] Zheng D, Dai Y, Wang S, Xing X (2015). MicroRNA-299-3p promotes the sensibility of lung cancer to doxorubicin through directly targeting ABCE1. Int J Clin Exp Pathol.

[47] Li X, Li X, Liao D, Wang X, Wu Z, Nie J, Bai M, Fu X, Mei Q, Han W (2015). Elevated microRNA-23a expression enhances the chemoresistance of colorectal cancer cells with microsatellite instability to 5-fluorouracil by directly targeting ABCF1. Curr Protein Pept Sci.

[48] Uhland K (2006). Matriptase and its putative role in cancer. Cell Mol Life Sci.

[49] Benaud CM, Oberst M, Dickson RB, Lin CY (2002). Deregulated activation of matriptase in breast cancer cells. Clin Exp Metastasis.

[50] Warren M, Twohig M, Pier T, Eickhoff J, Lin CY, Jarrard D, Huang W (2009). Protein expression of matriptase and its cognate inhibitor HAI-1 in human prostate cancer: a tissue microarray and automated quantitative analysis. Appl Immunohistochem Mol Morphol.

[51] Larive RM, Moriggi G, Menacho-Márquez M, Cañamero M, de Álava E, Alarcón B, Dosil M, Bustelo XR (2014). Contribution of the R-Ras2 GTP-binding protein to primary breast tumorigenesis and late-stage metastatic disease. Nat Commun.

[52] Luo H, Hao X, Ge C, Zhao F, Zhu M, Chen T, Yao M, He X, Li J (2010). TC21 promotes cell motility and metastasis by regulating the expression of E-cadherin and N-cadherin in hepatocellular carcinoma. Int J Oncol.

[53] Ritchie ME, Phipson B, Wu D, Hu Y, Law CW, Shi W, Smyth GK (2015). limma powers differential expression analyses for RNA-sequencing and microarray studies. Nucleic Acids Res.

[54] Weinberg RA (2014). The biology of cancer.

[55] Ohhashi S, Ohuchida K, Mizumoto K, Fujita H, Egami T, Yu J, Toma H, Sadatomi S, Nagai E (2008). TANAKA M: down-regulation of deoxycytidine kinase enhances acquired resistance to gemcitabine in pancreatic cancer. Anticancer Res.

[56] Lin A, Giuliano CJ, Palladino A, John KM, Abramowicz C, Yuan ML, Sausville EL, Lukow DA, Liu L, Chait AR et al. Off-target toxicity is a common mechanism of action of cancer drugs undergoing clinical trials. Sci Transl Med. 2019, 11(509). 10.1126/scitranslmed.aaw8412 PMC7717492 31511426

[57] Ellis LM, Hicklin DJ (2008). Pathways mediating resistance to vascular endothelial growth factor-targeted therapy. Clin Cancer Res.

[58] Sabnis AJ, Bivona TG (2019). Principles of resistance to targeted cancer therapy: lessons from basic and translational cancer biology. Trends Mol Med.

[59] Gottesman MM (2002). Mechanisms of cancer drug resistance. Annu Rev Med.

[60] Holohan C, Van Schaeybroeck S, Longley DB, Johnston PG (2013). Cancer drug resistance: an evolving paradigm. Nat Rev Cancer.

[61] McCall MN, Bolstad BM, Irizarry RA (2010). Frozen robust multiarray analysis (fRMA). Biostatistics.

[62] Mannheimer J, Fowles JS, Shaumberg K, Duval DL, Prasad A, Gustafson DL (2016). Abstract 1522: predicting drug sensitivity based on gene array data for cytotoxic chemotherapeutic agents. Can Res.

[63] Pedregosa F, Ga, #235, Varoquaux l, Gramfort A, Michel V, Thirion B, Grisel O, Blondel M, Prettenhofer P et al. Scikit-learn: machine learning in python. J Mach Learn Res. 2011, 12:2825–2830.

[64] Aric A. Hagber DAS, Pieter J. Swart: exploring netwrok structure, dynamics, and function using NetworkX. In: 7th Python science conference (SciPy2008): August 2008 2008; Pasadena, CA. 11–15.

