# Supplementary material for: Predicting chemosensitivity using drug perturbed gene dynamics
Source: BMC Bioinformatics. 2021 Jan 7;22:15. doi: 10.1186/s12859-020-03947-y (PMC7789515; doi:10.1186/s12859-020-03947-y)
Supplement: Supplementary file 1 — Additional file 1: Supplementary Materials. [file 12859_2020_3947_MOESM1_ESM.docx]

**Supplementary Materials**

**Combining DEGS:**

The best performing models without the inclusion of feature selection utilized 24 hour high dosed drug _gene_ expression (C_high_) performing marginally better than similar models using perturbed gene expression (1.2%). While, this was not a significant difference a possible explanation for the increase performance is that a signature from this dataset combined aspects from both the basal gene expression and gene perturbations. This presented an opportunity to select DEGS from two different data sets, basal gene expression and gene perturbations, and apply them to the gene expression of a single dataset hopefully capturing a more predictive signature.

The application of DEGs from both the basal data and the perturbed data outperformed models using a single set of DEGs from either the basal data or perturbed data as well as DEGs selected within the C_high_ dataset. The combination of 0nM and ΔC_high_ DEGs resulted in an average spearman correlation of 0.515 (Not including AZA) compared to 0.484 using ΔC_high_ DEGS, 0.396 using basal DEGs, and 0.476 using DEGs selected within (C_high_) the data; however, with the exception of basal data (p<0.0001), there performance of the other DEGs did not prove to be significant by a paired t-test. Nonetheless, for several drugs including bortezomib, doxorubicin, geldanamycin, paclitaxel, sorafenib, and sunitinib proved to outperform using any other DEG set most notably for bortezomib and sunitinib (Figure S1).


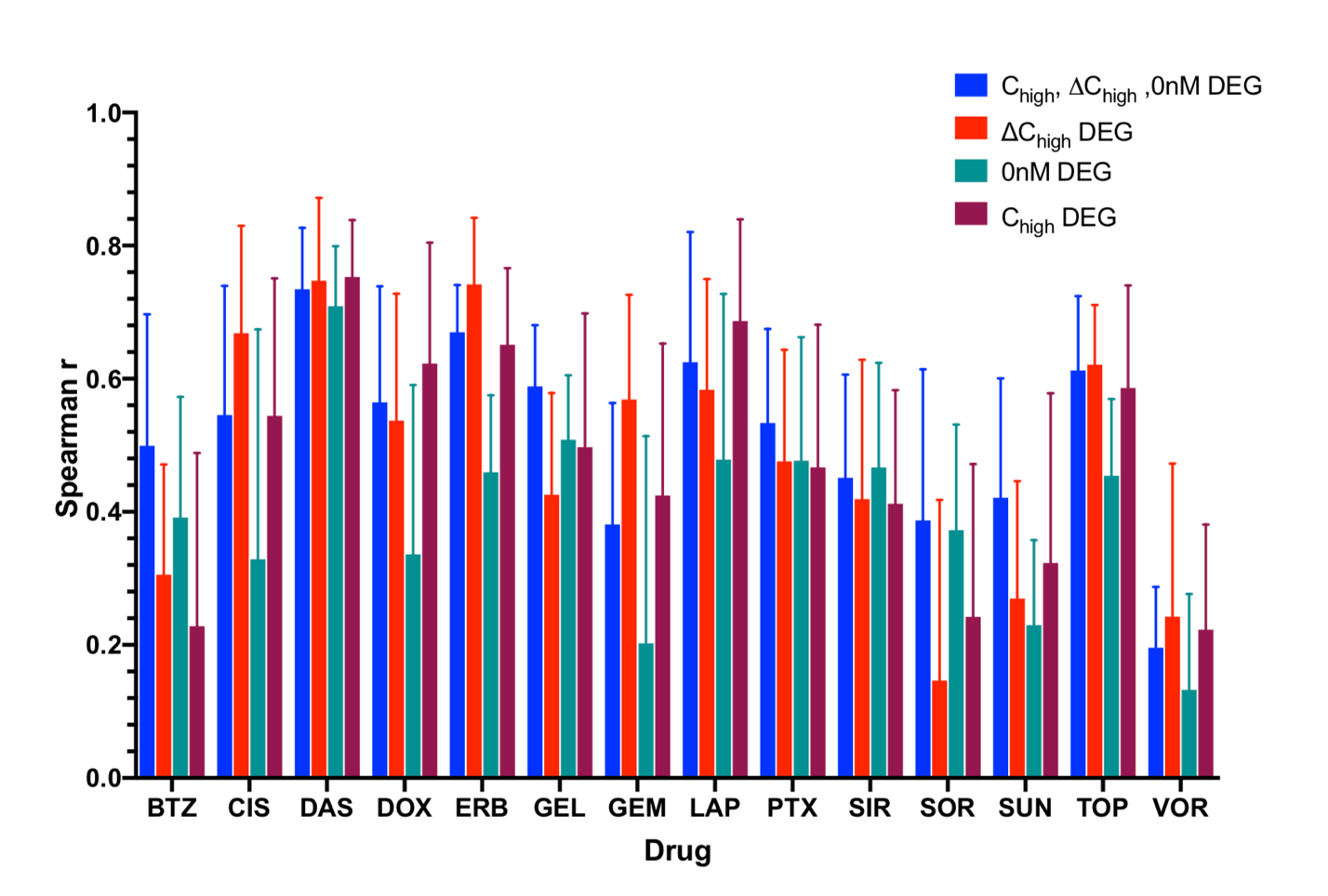


Figure S1. Models Built with DEGS from ΔC­_high_ and 0nM using C_high_ gene expression compared with other DEG Models

**Perturbation as Indicators of Drug response:**

One of the questions we sought to answer is how indicative is drug induced gene dynamics, specifically without the additional knowledge of drug mechanism or specific routes of resistance. How well can changes in gene expression alone predict drug response? Are predictive genes subject to greater dynamic perturbation? In order to ascertain the influence of we looked at the profile of DEGs, DEGs chosen from 0nM gene expression, and a 100 genes with the greatest magnitude of change in high dosed perturbation data. With the exception of sunitinib and lapatinib (%75,%83) the average magnitude of relative change between models using perturbed DEGs to basal DEGs was on average larger by %111 (SD=0.195) and a maximum of %160. For the 100 genes with the maximum magnitude of change were on average %247 (STDEV 0.45) larger ranging from a minimum of %181 and maximum %326 (figure S2 A). When looking at performance despite the fact that drug treatment resulted in a large magnitude of change very few of these genes are predictive of drug response resulting in a correlation of only 0.087 significantly lower than using random genes by %75. As referenced earlier, DEGs from high dosed perturbation performed %46.5 better then DEGs chosen from basal data (figure S2 B).


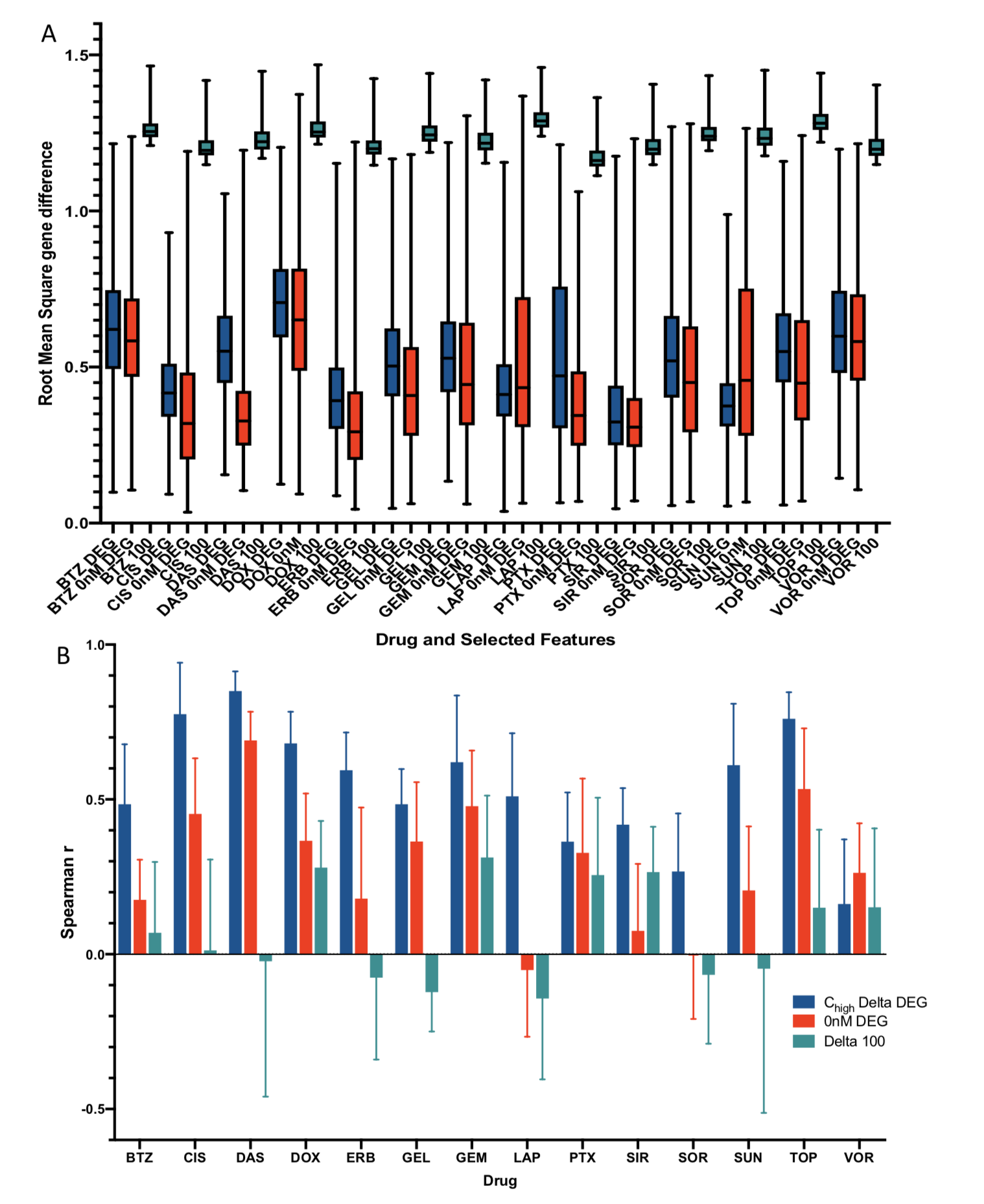


Figure S2: A. Distribution of the root mean squared magnitude of dynamic changes in gene expression after application of a high dose of drug in different gene sets. B. The performance using the respective DEGs for each drug.

Figure S4: Corresponding supplementary figure for figure 4 in text for A. Chigh and B. Clow

**Models Drug Related Genes**

In order to determine if gene expression of drug related genes at 0nM or ΔC_high_ were predictive the following genes were identified through the iLINCS web tool [1] and STITCH web tool [2] each drug

Table S1. Gene-drug interactions reported in the iLINCS and Stitch databases

| Drug | Genes |
| --- | --- |
| Bortezomib | PSMB1, PSMB2, PSMB6, TP53, MAPK8, CASP3, CYCS, JUN |
| Cisplatin | XIAP, TRAF1,TRAF2,LCK |
| Dasatinib | ABL1, FYN, LCK, KIT, YES1, EPHA2, LYN, PDGFRB, BCR, SRC, HCK |
| Doxorubicin | ABCB1, TOP2A, TP53, ABCG2, EFGR, CASP3, AKT1, MYC, ABCC1, ATM |
| Erlotinib | EFGR, EGF SLK, STAT3, CYP3A4, PTPN9 NOX4, AKT1, GAK,HGF |
| Geldanamycin | HSP90AA1, HSP90AB1, ERBB2, AKT1, HSP90B1, RAF1, HSPA4, DNAJB1, TRAP1, TP53 |
| Gemcitabine | RRM1 |
| Lapatinib | ERBB2, EGFR, ERBB3, ERBB4, ESR1, AKT1, VEGFA, ABBC10,MCL1, TP53 |
| Paclitaxel | AURKB, CDK1, MAPRE3 TNF, VEGA, MMP2, CYCS, CAMKMT, CDH1, WNTSA, TUBB, NR1I2 |
| Sirolimus | MTOR, FKBP1A |
| Sorafenib | RET, BRAF, FLT3, KDR, RAF1, FLT1, FLT4, PDGFRA, CSF1R, AXL, RPS6KB1 |
| Sunitinib | PDGFRB, KIT, FLT3, KDR, FLT1, FTL4, PDGFRA, CSF1R, AXL, RPS6KB1 |
| Topotecan | TOP1 |
| Vorinostat | HDAC1,HDAC2,HDAC3,HDAC6, HDAC8, TP53, K2AFX, BCL2L1, HDAC7, HSP90AA1 |

Compared to DEG models for both ΔC­_high_ and 0nM DEGS the performance of models using the genes above was significantly worse (figure S3).


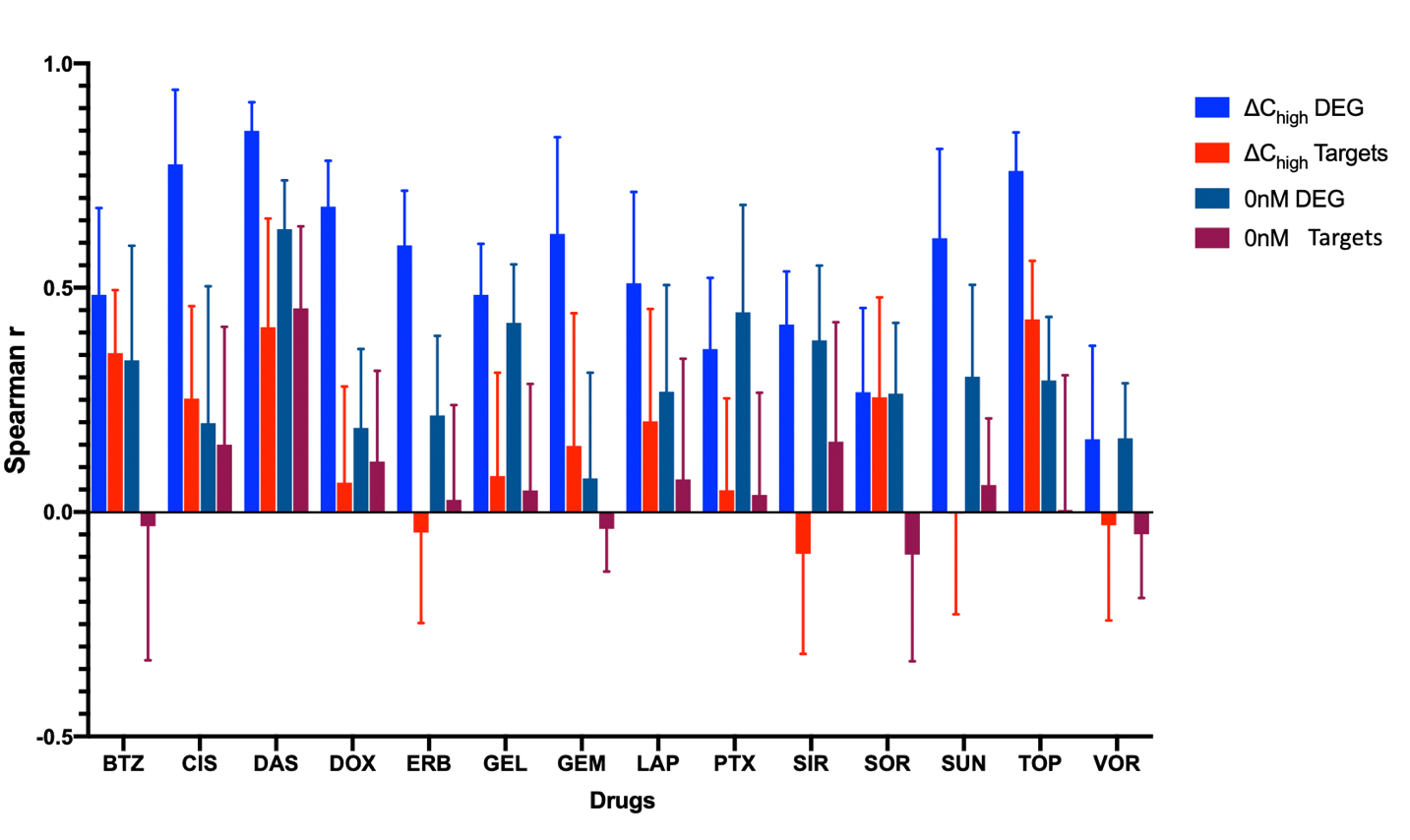


Figure S3: Models using genes defined in Table S1 compared to Models using DEGs from different gene expression profiles.

Figure S4: Corresponding supplementary figure for figure 4 in text for A. C_high_ and B. C_low_

**Additional Genes From Network Analysis**

Table S2. Additional genes with top clique participation for ΔC_high­_ DEGs

| Bortezomib | Developmentally regulated GTP binding protein 2 (DRG2)[3]  MicroRNA 939 MIR939 [4]  Cell division cycle 37 (CDC37) [5, 6]  Eukaryotic translation initiation factor 3 subunit G (EIF3G) [7]  Squamous cell carcinoma antigen recognized by T-cells 1 (SART1) |
| --- | --- |
| Cisplatin | SWI/SNF related, matrix associated, actin dependent regulator of chromatin subfamily c member 1 (SMARCC1) [8, 9]  Drosha ribonuclease III (DROSHA) [10, 11]  Nuclear receptor coactivator (NCOA1, SRC1) [12]  ADAM metallopeptidase domain 10 (ADAM10) [13]  Tetratricopeptide repeat domain 28 (TTC28) [14] |
| Doxorubicin | Heterogenous nuclear ribonucleoprotein A0 (HNRNPA0) [15]  Eukaryotic translation initiation factor 4E (EIF4E) [16-18]  Cytokine receptor like factor3 (CRLF3, p48.2) [19]  MOB family member 4, phocein (MOB4) [20] |
| Erlotinib | survivin (BIRC5) [21-23]  H2A histone family member X(H2AX) [24]  BUB2 mitotic checkpoint serine/threonine kinase B(BUB1B) [25, 26] |
| Geldanamycin | Proteasome activator subunit 3 (PSME3) [27, 28]  Proliferation-associated 2G4 (PA2G4)(EBP1) [29, 30]  RAN binding protein 1(RANBP1)[31, 32]  Cell division cycle 25A (CDC25A) [33-35]  PBX homeobox interacting protein (PBXIP1,HPIP)[36, 37] |
| Gemcitabine | Peroxiredoxin 1 (PRDX1) [38, 39]  BUD31 homolog (BUD31) [40]  DEAD-box helicase 27 (DDX27)[41]  Small ubiquitin-like modifier 3 (SUMO3) [42]  Tyrosine 3-monooxygenase/tryptophan 5-monooxygenase activation protein epsilon (YWHAE, 14-3-3 epsilon) [43, 44] |
| Lapatinib | Tumor susceptibility 101 (TSG101) [45, 46]  Chromosome segregation 1 like (CSE1L) [47, 48]  TIMELESS interacting protein (TIPIN) [49]  NME/NM23 nucleoside diphosphate kinase1 (NME1) [50-52]  GINS complex subunit 2 (GINS2)[53-55] |
| Paclitaxel | RNA binding motif protein 5(RBM5,LUCA15)[56-58]  Topoisomerase DNA II binding protein (TOPBP1) [59-61]  BUB3, mitotic checkpoint protein (BUB3)[62]  SET domain containing 1B (SETD1B) [63]  Histone cluster 4 H4(HIST4H4) [64, 65] |
| Sirolimus | Ubiquitin associated protein 2 like (UBA2P2L) [66, 67]  RE1 Silencing transcription factor (REST) [68, 69] |
| Sorafenib | Proteasome activator subunit 3(PSME3)[27, 70]  Heterogeneous nuclear ribonucleoprotein A/B (HNRNPAB)[71]  Chaperonin containing TCP1 subunit 2 (CCT2) [72, 73] |
| Sunitinib | Eukaryotic translation initiation factor 2 subunit alpha (EIF2S1)[74, 75]  Calcyclin binding protein (CACYBP) [76, 77]  G1 to S phase transition 1 (GSPT1) [78, 79]  DExD-box helicase 39A (DDX39A) [80, 81]  Proliferation-associated 2G4 (PA2G4) [30, 82] |
| Topotecan | RAD23 homolog B. nucleotide excision repair protein (RAD23B) [83]  Cullin 4A (CUL4A) [84, 85]  BUD31 homolog BUD31 [40]  Complement C1q binding protein C1qBP [86, 87]  Methionyl aminopeptidase 2 (METAP2) [88-90] |
| Vorinostat | Eukaryotic translation elongation factor 1 epsilon (AIMP3) [91]  NOP16 nucleolar protein (NOP16, HSPC111) [92]  Proliferation-associated 2G4 (PA2G4,EBP1) [29, 30, 93, 94] |

Table S3. Genes with top Clique Participation for 0nM DEGs

| Bortezomib | Protein phosphatase 2 scaffold subunit Abeta (PPP2R1B) [95, 96]  MAD2 mitotic arrest deficient-like MAD2L1 [97-99]  Polo like kinase 4 (PLK4,SAK) [100, 101] |
| --- | --- |
| Cisplatin | Epithelial cell adhesion molecule (EPCAM) [102-104]  Suppression of tumorigenicity 14 (ST14, TADG15) [105]  Zinc-Finger protein 165(ZNF165)[106]  Serine peptidase inhibitor, Kunitz type 2 (SPINT2)[107, 108]  F11 receptor (F11R, JAM-A) [109, 110] |
| Doxorubicin | Tyrosine 3-monooxygenase/tryptophan 5-monooxygenenase activation protein zeta (YWHAZ, 14-3-3 Zeta) [111-113]  Centrosomal protein 57 (CEP57)[114]  Fli-1 proto-oncogene, ETS transcription factor (FLI1) [115, 116]  Ribosomal protein S4, X-linked(RPS4X) [117, 118] |
| Erlotinib | Myosin light chain kinase (MYLK) [119, 120]  N-myristoyltransferase 2(NMT2) [121]  Related RAS viral (r-ras) oncogene homolog 2(RRAS2,TC21)[122-124]  NOP14 nucleolar protein (NOP14) [125] |
| Geldanamycin | GLI pathogenesis related 1(GLIPR1) [126, 127]  NUAK family kinase(NUAK1) [128-130]  Microtubule associated protein 1B (MAP1B) [131]  microRNA 22(MIR22) [132, 133]  FOS like 2, AP-1 transcription factor subunit (FOSL2, FRA2) [134, 135] |
| Gemcitabine | Cadherin 1 (CDH1) [136, 137]  CCCTC-binding factor (CTCF) [138, 139]  GINS complex subunit 3(GINS3,PSF3) [140] |
| Lapatinib | RAP1 GTPase activating protein (RAP1GAP) [141-143]  Transforming growth factor alpha (TGFA) [144, 145]  Lysophosphatidic acid receptor 2 (LPAR2) [146, 147]  Tissue factor pathway inhibitor 2 (TFPI2)[148]  F11 receptor(F11R,JAM-A) [109, 110] |
| Paclitaxel | Serine and arginine rich splicing factor 2 (SRSF2) [149, 150]  Cell division cycle 25A (CDC25A) [34, 35, 151] |
| Sirolimus | Nucleolin (NCL) [152]  Serine and arginine rich splicing factor 2 (SRSF2) [149, 150]  microRNA 1244-1(MIR1244-1) [153]  polo like kinase 4 (PLK4,SAK) [154, 155]  checkpoint kinase 1(CHEK1) [156, 157] |
| Sorafenib | Tropomyosin 1 (alpha) (TPM1) [158, 159]  CD24 molecule (CD24)[103, 160, 161]  E74 like ETS transcription factor 3 (ELF3) [162, 163]  Caspase recruitment domain family member 10 (CARD10,CARMA3)[164, 165] |
| Sunitinib | Cell division cycle 25A (CDC25A) [35, 151, 166]  Cyclin dependent kinase 8 (CDK8) [167, 168]  Squamous cell carcinoma antigen recognized by T-cells 3(SART3)[169, 170]  NOP14 nucleolar protein (NOP14)[125]  PLAG1 like zinc finger 2(PLAGL2) [171-173] |
| Topotecan | Cadherin 1 (CDH1)[122, 137]  E74 like ETS transcription factor 3 (ELF3)[162, 163, 174]  P21 (RAC1) activated kinase 6 (PAK6)[175, 176]  Microtubule associated protein 7(MAP7) [177] |
| Vorinostat | Plasaminogen activator, urokinase (PLAU) [178]  Microtubule associated monooxygenase, calponin and LIM domain containing 2 (MICAL2)[179]  Transforming growth factor beta2 (TGFB2) [180, 181]  Annexin II (ANXA2) [82, 182]  Neuropilin 1 (NRP1)[183] |

References Cited

1. Pilarczyk M, Najafabadi MF, Kouril M, Vasiliauskas J, Niu W, Shamsaei B, Mahi N, Zhang L, Clark N, Ren Y *et al*: **Connecting omics signatures of diseases, drugs, and mechanisms of actions with iLINCS**. *bioRxiv* 2019:826271.

2. Szklarczyk D, Santos A, von Mering C, Jensen LJ, Bork P, Kuhn M: **STITCH 5: augmenting protein-chemical interaction networks with tissue and affinity data**. *Nucleic Acids Res* 2016, **44**(D1):D380-384.

3. Xu C, Li H, Zhang L, Jia T, Duan L, Lu C: **MicroRNA‑1915‑3p prevents the apoptosis of lung cancer cells by downregulating DRG2 and PBX2**. *Mol Med Rep* 2016, **13**(1):505-512.

4. Ying X, Li-ya Q, Feng Z, Yin W, Ji-hong L: **MiR-939 promotes the proliferation of human ovarian cancer cells by repressing APC2 expression**. *Biomed Pharmacother* 2015, **71**:64-69.

5. Wu F, Peacock SO, Rao S, Lemmon SK, Burnstein KL: **Novel interaction between the co-chaperone Cdc37 and Rho GTPase exchange factor Vav3 promotes androgen receptor activity and prostate cancer growth**. *J Biol Chem* 2013, **288**(8):5463-5474.

6. Basso AD, Solit DB, Chiosis G, Giri B, Tsichlis P, Rosen N: **Akt forms an intracellular complex with heat shock protein 90 (Hsp90) and Cdc37 and is destabilized by inhibitors of Hsp90 function**. *J Biol Chem* 2002, **277**(42):39858-39866.

7. Kim JT, Lee SJ, Kim BY, Lee CH, Yeom YI, Choe YK, Yoon DY, Chae SK, Kim JW, Yang Y *et al*: **Caspase-mediated cleavage and DNase activity of the translation initiation factor 3, subunit G (eIF3g)**. *FEBS Lett* 2013, **587**(22):3668-3674.

8. Heebøll S, Borre M, Ottosen PD, Andersen CL, Mansilla F, Dyrskjøt L, Orntoft TF, Tørring N: **SMARCC1 expression is upregulated in prostate cancer and positively correlated with tumour recurrence and dedifferentiation**. *Histol Histopathol* 2008, **23**(9):1069-1076.

9. Andersen CL, Christensen LL, Thorsen K, Schepeler T, Sørensen FB, Verspaget HW, Simon R, Kruhøffer M, Aaltonen LA, Laurberg S *et al*: **Dysregulation of the transcription factors SOX4, CBFB and SMARCC1 correlates with outcome of colorectal cancer**. *Br J Cancer* 2009, **100**(3):511-523.

10. Zhang H, Hou Y, Xu L, Zeng Z, Wen S, Du YE, Sun K, Yin J, Lang L, Tang X *et al*: **Cytoplasmic Drosha Is Aberrant in Precancerous Lesions of Gastric Carcinoma and Its Loss Predicts Worse Outcome for Gastric Cancer Patients**. *Dig Dis Sci* 2016, **61**(4):1080-1090.

11. Zhou J, Cai J, Huang Z, Ding H, Wang J, Jia J, Zhao Y, Huang D, Wang Z: **Proteomic identification of target proteins following Drosha knockdown in cervical cancer**. *Oncol Rep* 2013, **30**(5):2229-2237.

12. Wang L, Yu Y, Chow DC, Yan F, Hsu CC, Stossi F, Mancini MA, Palzkill T, Liao L, Zhou S *et al*: **Characterization of a Steroid Receptor Coactivator Small Molecule Stimulator that Overstimulates Cancer Cells and Leads to Cell Stress and Death**. *Cancer Cell* 2015, **28**(2):240-252.

13. Liu S, Zhang W, Liu K, Ji B, Wang G: **Silencing ADAM10 inhibits the in vitro and in vivo growth of hepatocellular carcinoma cancer cells**. *Mol Med Rep* 2015, **11**(1):597-602.

14. Izumiyama T, Minoshima S, Yoshida T, Shimizu N: **A novel big protein TPRBK possessing 25 units of TPR motif is essential for the progress of mitosis and cytokinesis**. *Gene* 2012, **511**(2):202-217.

15. Cannell IG, Merrick KA, Morandell S, Zhu CQ, Braun CJ, Grant RA, Cameron ER, Tsao MS, Hemann MT, Yaffe MB: **A Pleiotropic RNA-Binding Protein Controls Distinct Cell Cycle Checkpoints to Drive Resistance of p53-Defective Tumors to Chemotherapy**. *Cancer Cell* 2015, **28**(5):623-637.

16. Hoang B, Benavides A, Shi Y, Yang Y, Frost P, Gera J, Lichtenstein A: **The PP242 mammalian target of rapamycin (mTOR) inhibitor activates extracellular signal-regulated kinase (ERK) in multiple myeloma cells via a target of rapamycin complex 1 (TORC1)/eukaryotic translation initiation factor 4E (eIF-4E)/RAF pathway and activation is a mechanism of resistance**. *J Biol Chem* 2012, **287**(26):21796-21805.

17. Wheater MJ, Johnson PW, Blaydes JP: **The role of MNK proteins and eIF4E phosphorylation in breast cancer cell proliferation and survival**. *Cancer Biol Ther* 2010, **10**(7):728-735.

18. Muta D, Makino K, Nakamura H, Yano S, Kudo M, Kuratsu J: **Inhibition of eIF4E phosphorylation reduces cell growth and proliferation in primary central nervous system lymphoma cells**. *J Neurooncol* 2011, **101**(1):33-39.

19. Yang F, Xu YP, Li J, Duan SS, Fu YJ, Zhang Y, Zhao Y, Qiao WT, Chen QM, Geng YQ *et al*: **Cloning and characterization of a novel intracellular protein p48.2 that negatively regulates cell cycle progression**. *Int J Biochem Cell Biol* 2009, **41**(11):2240-2250.

20. Tang F, Zhang L, Xue G, Hynx D, Wang Y, Cron PD, Hundsrucker C, Hergovich A, Frank S, Hemmings BA *et al*: **hMOB3 modulates MST1 apoptotic signaling and supports tumor growth in glioblastoma multiforme**. *Cancer Res* 2014, **74**(14):3779-3789.

21. Rödel C, Haas J, Groth A, Grabenbauer GG, Sauer R, Rödel F: **Spontaneous and radiation-induced apoptosis in colorectal carcinoma cells with different intrinsic radiosensitivities: survivin as a radioresistance factor**. *Int J Radiat Oncol Biol Phys* 2003, **55**(5):1341-1347.

22. Zaffaroni N, Pennati M, Colella G, Perego P, Supino R, Gatti L, Pilotti S, Zunino F, Daidone MG: **Expression of the anti-apoptotic gene survivin correlates with taxol resistance in human ovarian cancer**. *Cell Mol Life Sci* 2002, **59**(8):1406-1412.

23. Lee JP, Chang KH, Han JH, Ryu HS: **Survivin, a novel anti-apoptosis inhibitor, expression in uterine cervical cancer and relationship with prognostic factors**. *Int J Gynecol Cancer* 2005, **15**(1):113-119.

24. Strasberg Rieber M, Viola-Rhenals M, Rieber M: **Attenuation of genotoxicity under adhesion-restrictive conditions through modulation of p53, gamma H2AX and nuclear DNA organization**. *Apoptosis* 2007, **12**(2):449-458.

25. Ikawa-Yoshida A, Ando K, Oki E, Saeki H, Kumashiro R, Taketani K, Ida S, Tokunaga E, Kitao H, Morita M *et al*: **Contribution of BubR1 to oxidative stress-induced aneuploidy in p53-deficient cells**. *Cancer Med* 2013, **2**(4):447-456.

26. Fragoso MC, Almeida MQ, Mazzuco TL, Mariani BM, Brito LP, Gonçalves TC, Alencar GA, Lima Lde O, Faria AM, Bourdeau I *et al*: **Combined expression of BUB1B, DLGAP5, and PINK1 as predictors of poor outcome in adrenocortical tumors: validation in a Brazilian cohort of adult and pediatric patients**. *Eur J Endocrinol* 2012, **166**(1):61-67.

27. Li J, Feng X, Sun C, Zeng X, Xie L, Xu H, Li T, Wang R, Xu X, Zhou X *et al*: **Associations between proteasomal activator PA28γ and outcome of oral squamous cell carcinoma: Evidence from cohort studies and functional analyses**. *EBioMedicine* 2015, **2**(8):851-858.

28. Xu X, Liu D, Ji N, Li T, Li L, Jiang L, Li J, Zhang P, Zeng X, Chen Q: **A novel transcript variant of proteasome activator 28γ: Identification and function in oral cancer cells**. *Int J Oncol* 2015, **47**(1):188-194.

29. Zhang F, Liu Y, Wang Z, Sun X, Yuan J, Wang T, Tian R, Ji W, Yu M, Zhao Y *et al*: **A novel Anxa2-interacting protein Ebp1 inhibits cancer proliferation and invasion by suppressing Anxa2 protein level**. *Molecular and Cellular Endocrinology* 2015, **411**:75-85.

30. Zhang Y, Akinmade D, Hamburger AW: **Inhibition of heregulin mediated MCF-7 breast cancer cell growth by the ErbB3 binding protein EBP1**. *Cancer Lett* 2008, **265**(2):298-306.

31. Amato R, Scumaci D, D'Antona L, Iuliano R, Menniti M, Di Sanzo M, Faniello MC, Colao E, Malatesta P, Zingone A *et al*: **Sgk1 enhances RANBP1 transcript levels and decreases taxol sensitivity in RKO colon carcinoma cells**. *Oncogene* 2013, **32**(38):4572-4578.

32. Rensen WM, Roscioli E, Tedeschi A, Mangiacasale R, Ciciarello M, Di Gioia SA, Lavia P: **RanBP1 downregulation sensitizes cancer cells to taxol in a caspase-3-dependent manner**. *Oncogene* 2009, **28**(15):1748-1758.

33. Lin TC, Lin PL, Cheng YW, Wu TC, Chou MC, Chen CY, Lee H: **MicroRNA-184 Deregulated by the MicroRNA-21 Promotes Tumor Malignancy and Poor Outcomes in Non-small Cell Lung Cancer via Targeting CDC25A and c-Myc**. *Ann Surg Oncol* 2015, **22 Suppl 3**:S1532-1539.

34. Li N, Zhong X, Lin X, Guo J, Zou L, Tanyi JL, Shao Z, Liang S, Wang L-P, Hwang W-T *et al*: **Lin-28 homologue A (LIN28A) promotes cell cycle progression via regulation of cyclin-dependent kinase 2 (CDK2), cyclin D1 (CCND1), and cell division cycle 25 homolog A (CDC25A) expression in cancer**. *J Biol Chem* 2012, **287**(21):17386-17397.

35. Chiu Y-T, Han H-Y, Leung SC-L, Yuen H-F, Chau C-W, Guo Z, Qiu Y, Chan K-W, Wang X, Wong Y-C *et al*: **CDC25A Functions as a Novel Ar Corepressor in Prostate Cancer Cells**. *Journal of Molecular Biology* 2009, **385**(2):446-456.

36. van Vuurden DG, Aronica E, Hulleman E, Wedekind LE, Biesmans D, Malekzadeh A, Bugiani M, Geerts D, Noske DP, Vandertop WP *et al*: **Pre-B-cell leukemia homeobox interacting protein 1 is overexpressed in astrocytoma and promotes tumor cell growth and migration**. *Neuro Oncol* 2014, **16**(7):946-959.

37. Okada S, Irié T, Tanaka J, Yasuhara R, Yamamoto G, Isobe T, Hokazono C, Tachikawa T, Kohno Y, Mishima K: **Potential role of hematopoietic pre-B-cell leukemia transcription factor-interacting protein in oral carcinogenesis**. *J Oral Pathol Med* 2015, **44**(2):115-125.

38. Song IS, Kim SU, Oh NS, Kim J, Yu DY, Huang SM, Kim JM, Lee DS, Kim NS: **Peroxiredoxin I contributes to TRAIL resistance through suppression of redox-sensitive caspase activation in human hepatoma cells**. *Carcinogenesis* 2009, **30**(7):1106-1114.

39. Dey KK, Pal I, Bharti R, Dey G, Kumar BN, Rajput S, Parekh A, Parida S, Halder P, Kulavi I *et al*: **Identification of RAB2A and PRDX1 as the potential biomarkers for oral squamous cell carcinoma using mass spectrometry-based comparative proteomic approach**. *Tumour Biol* 2015, **36**(12):9829-9837.

40. Xu W, Huang H, Yu L, Cao L: **Meta-analysis of gene expression profiles indicates genes in spliceosome pathway are up-regulated in hepatocellular carcinoma (HCC)**. *Med Oncol* 2015, **32**(4):96.

41. Zhou J, Yong WP, Yap CS, Vijayaraghavan A, Sinha RA, Singh BK, Xiu S, Manesh S, Ngo A, Lim A *et al*: **An integrative approach identified genes associated with drug response in gastric cancer**. *Carcinogenesis* 2015, **36**(4):441-451.

42. Liu J, Sha M, Wang Q, Ma Y, Geng X, Gao Y, Feng L, Shen Y, Shen Y: **Small ubiquitin-related modifier 2/3 interacts with p65 and stabilizes it in the cytoplasm in HBV-associated hepatocellular carcinoma**. *BMC Cancer* 2015, **15**:675.

43. Ko BS, Chang TC, Hsu C, Chen YC, Shen TL, Chen SC, Wang J, Wu KK, Jan YJ, Liou JY: **Overexpression of 14-3-3ε predicts tumour metastasis and poor survival in hepatocellular carcinoma**. *Histopathology* 2011, **58**(5):705-711.

44. Konishi H, Nakagawa T, Harano T, Mizuno K, Saito H, Masuda A, Matsuda H, Osada H, Takahashi T: **Identification of frequent G(2) checkpoint impairment and a homozygous deletion of 14-3-3epsilon at 17p13.3 in small cell lung cancers**. *Cancer Res* 2002, **62**(1):271-276.

45. Zhang Y, Song M, Cui ZS, Li CY, Xue XX, Yu M, Lu Y, Zhang SY, Wang EH, Wen YY: **Down-regulation of TSG101 by small interfering RNA inhibits the proliferation of breast cancer cells through the MAPK/ERK signal pathway**. *Histol Histopathol* 2011, **26**(1):87-94.

46. Gu RJ, Wang SC, Sun G, Zhuang BW, Liu DL: **[Expression and significance of tumor susceptibility gene 101 in hepatocellular carcinoma tissues]**. *Xi Bao Yu Fen Zi Mian Yi Xue Za Zhi* 2012, **28**(7):738-740.

47. Yuksel UM, Turker I, Dilek G, Dogan L, Gulcelik MA, Oksuzoglu B: **Does CSE1L Overexpression Affect Distant Metastasis Development in Breast Cancer?** *Oncol Res Treat* 2015, **38**(9):431-434.

48. Shiraki K, Fujikawa K, Sugimoto K, Ito T, Yamanaka T, Suzuki M, Yoneda K, Sugimoto K, Takase K, Nakano T: **Cellular apoptosis susceptibility protein and proliferation in human hepatocellular carcinoma**. *Int J Mol Med* 2006, **18**(1):77-81.

49. Baldeyron C, Brisson A, Tesson B, Némati F, Koundrioukoff S, Saliba E, De Koning L, Martel E, Ye M, Rigaill G *et al*: **TIPIN depletion leads to apoptosis in breast cancer cells**. *Mol Oncol* 2015, **9**(8):1580-1598.

50. Tomita M, Ayabe T, Matsuzaki Y, Edagawa M, Maeda M, Shimizu T, Hara M, Onitsuka T: **Expression of nm23-H1 gene product in esophageal squamous cell carcinoma and its association with vessel invasion and survival**. *BMC Cancer* 2001, **1**:3.

51. Galani E, Sgouros J, Petropoulou C, Janinis J, Aravantinos G, Dionysiou-Asteriou D, Skarlos D, Gonos E: **Correlation of MDR-1, nm23-H1 and H Sema E gene expression with histopathological findings and clinical outcome in ovarian and breast cancer patients**. *Anticancer Res* 2002, **22**(4):2275-2280.

52. Kushlinskii NE, Delektorskaya VV, Mochal'nikova VV, Sini L, Yurchenko AA, Ryabov AB, Stilidi IS: **Analysis of NM23 protein and components of plasminogen activation system in tumors of patients with stomach cancer with consideration for disease clinical picture and morphology**. *Bull Exp Biol Med* 2008, **146**(6):786-790.

53. Rantala JK, Edgren H, Lehtinen L, Wolf M, Kleivi K, Vollan HKM, Aaltola A-R, Laasola P, Kilpinen S, Saviranta P *et al*: **Integrative functional genomics analysis of sustained polyploidy phenotypes in breast cancer cells identifies an oncogenic profile for GINS2**. *Neoplasia* 2010, **12**(11):877-888.

54. Gao Y, Wang S, Liu B, Zhong L: **Roles of GINS2 in K562 human chronic myelogenous leukemia and NB4 acute promyelocytic leukemia cells**. *Int J Mol Med* 2013, **31**(6):1402-1410.

55. Zheng M, Zhou Y, Yang X, Tang J, Wei D, Zhang Y, Jiang J-L, Chen Z-n, Zhu P: **High GINS2 transcript level predicts poor prognosis and correlates with high histological grade and endocrine therapy resistance through mammary cancer stem cells in breast cancer patients**. *Breast Cancer Research and Treatment* 2014, **148**(2):423-436.

56. Rintala-Maki ND, Abrasonis V, Burd M, Sutherland LC: **Genetic instability of RBM5/LUCA-15/H37 in MCF-7 breast carcinoma sublines may affect susceptibility to apoptosis**. *Cell Biochem Funct* 2004, **22**(5):307-313.

57. Kobayashi T, Ishida J, Musashi M, Ota S, Yoshida T, Shimizu Y, Chuma M, Kawakami H, Asaka M, Tanaka J *et al*: **p53 transactivation is involved in the antiproliferative activity of the putative tumor suppressor RBM5**. *Int J Cancer* 2011, **128**(2):304-318.

58. Oh JJ, Taschereau EO, Koegel AK, Ginther CL, Rotow JK, Isfahani KZ, Slamon DJ: **RBM5/H37 tumor suppressor, located at the lung cancer hot spot 3p21.3, alters expression of genes involved in metastasis**. *Lung Cancer* 2010, **70**(3):253-262.

59. Liu K, Bellam N, Lin HY, Wang B, Stockard CR, Grizzle WE, Lin WC: **Regulation of p53 by TopBP1: a potential mechanism for p53 inactivation in cancer**. *Mol Cell Biol* 2009, **29**(10):2673-2693.

60. Yamane K, Chen J, Kinsella TJ: **Both DNA topoisomerase II-binding protein 1 and BRCA1 regulate the G2-M cell cycle checkpoint**. *Cancer Res* 2003, **63**(12):3049-3053.

61. Forma E, Wójcik-Krowiranda K, Jóźwiak P, Szymczyk A, Bieńkiewicz A, Bryś M, Krześlak A: **Topoisomerase IIβ binding protein 1 c.*229C>T (rs115160714) gene polymorphism and endometrial cancer risk**. *Pathol Oncol Res* 2014, **20**(3):597-602.

62. Yoon YM, Baek KH, Jeong SJ, Shin HJ, Ha GH, Jeon AH, Hwang SG, Chun JS, Lee CW: **WD repeat-containing mitotic checkpoint proteins act as transcriptional repressors during interphase**. *FEBS Lett* 2004, **575**(1-3):23-29.

63. Choi YJ, Oh HR, Choi MR, Gwak M, An CH, Chung YJ, Yoo NJ, Lee SH: **Frameshift mutation of a histone methylation-related gene SETD1B and its regional heterogeneity in gastric and colorectal cancers with high microsatellite instability**. *Hum Pathol* 2014, **45**(8):1674-1681.

64. Boix-Chornet M, Fraga MF, Villar-Garea A, Caballero R, Espada J, Nuñez A, Casado J, Largo C, Casal JI, Cigudosa JC *et al*: **Release of hypoacetylated and trimethylated histone H4 is an epigenetic marker of early apoptosis**. *J Biol Chem* 2006, **281**(19):13540-13547.

65. Marquard L, Gjerdrum LM, Christensen IJ, Jensen PB, Sehested M, Ralfkiaer E: **Prognostic significance of the therapeutic targets histone deacetylase 1, 2, 6 and acetylated histone H4 in cutaneous T-cell lymphoma**. *Histopathology* 2008, **53**(3):267-277.

66. Li D, Huang Y: **Knockdown of ubiquitin associated protein 2-like inhibits the growth and migration of prostate cancer cells**. *Oncol Rep* 2014, **32**(4):1578-1584.

67. Zhao B, Zong G, Xie Y, Li J, Wang H, Bian E: **Downregulation of ubiquitin-associated protein 2-like with a short hairpin RNA inhibits human glioma cell growth in vitro**. *Int J Mol Med* 2015, **36**(4):1012-1018.

68. Zhou Z, Yu L, Kleinerman ES: **EWS-FLI-1 regulates the neuronal repressor gene REST, which controls Ewing sarcoma growth and vascular morphology**. *Cancer* 2014, **120**(4):579-588.

69. Huang Z, Bao S: **Ubiquitination and deubiquitination of REST and its roles in cancers**. *FEBS Lett* 2012, **586**(11):1602-1605.

70. Chen D, Yang X, Huang L, Chi P: **The expression and clinical significance of PA28 γ in colorectal cancer**. *J Investig Med* 2013, **61**(8):1192-1196.

71. Zhou ZJ, Dai Z, Zhou SL, Hu ZQ, Chen Q, Zhao YM, Shi YH, Gao Q, Wu WZ, Qiu SJ *et al*: **HNRNPAB induces epithelial-mesenchymal transition and promotes metastasis of hepatocellular carcinoma by transcriptionally activating SNAIL**. *Cancer Res* 2014, **74**(10):2750-2762.

72. Zou Q, Yang ZL, Yuan Y, Li JH, Liang LF, Zeng GX, Chen SL: **Clinicopathological features and CCT2 and PDIA2 expression in gallbladder squamous/adenosquamous carcinoma and gallbladder adenocarcinoma**. *World J Surg Oncol* 2013, **11**:143.

73. Guest ST, Kratche ZR, Bollig-Fischer A, Haddad R, Ethier SP: **Two members of the TRiC chaperonin complex, CCT2 and TCP1 are essential for survival of breast cancer cells and are linked to driving oncogenes**. *Exp Cell Res* 2015, **332**(2):223-235.

74. Rajesh K, Krishnamoorthy J, Kazimierczak U, Tenkerian C, Papadakis AI, Wang S, Huang S, Koromilas AE: **Phosphorylation of the translation initiation factor eIF2α at serine 51 determines the cell fate decisions of Akt in response to oxidative stress**. *Cell Death Dis* 2015, **6**(1):e1591.

75. Tuval-Kochen L, Paglin S, Keshet G, Lerenthal Y, Nakar C, Golani T, Toren A, Yahalom J, Pfeffer R, Lawrence Y: **Eukaryotic initiation factor 2α--a downstream effector of mammalian target of rapamycin--modulates DNA repair and cancer response to treatment**. *PLoS One* 2013, **8**(10):e77260.

76. Fu C, Wan Y, Shi H, Gong Y, Wu Q, Yao Y, Niu M, Li Z, Xu K: **Expression and regulation of CacyBP/SIP in chronic lymphocytic leukemia cell balances of cell proliferation with apoptosis**. *J Cancer Res Clin Oncol* 2016, **142**(4):741-748.

77. Zhai HH, Meng J, Wang JB, Liu ZX, Li YF, Feng SS: **CacyBP/SIP nuclear translocation induced by gastrin promotes gastric cancer cell proliferation**. *World J Gastroenterol* 2014, **20**(29):10062-10070.

78. Lee JA, Park JE, Lee DH, Park SG, Myung PK, Park BC, Cho S: **G1 to S phase transition protein 1 induces apoptosis signal-regulating kinase 1 activation by dissociating 14-3-3 from ASK1**. *Oncogene* 2008, **27**(9):1297-1305.

79. Malta-Vacas J, Chauvin C, Gonçalves L, Nazaré A, Carvalho C, Monteiro C, Bagrel D, Jean-Jean O, Brito M: **eRF3a/GSPT1 12-GGC allele increases the susceptibility for breast cancer development**. *Oncol Rep* 2009, **21**(6):1551-1558.

80. Kato M, Wei M, Yamano S, Kakehashi A, Tamada S, Nakatani T, Wanibuchi H: **DDX39 acts as a suppressor of invasion for bladder cancer**. *Cancer Sci* 2012, **103**(7):1363-1369.

81. Sugiura T, Nagano Y, Noguchi Y: **DDX39, upregulated in lung squamous cell cancer, displays RNA helicase activities and promotes cancer cell growth**. *Cancer Biol Ther* 2007, **6**(6):957-964.

82. Zhang F, Liu Y, Wang Z, Sun X, Yuan J, Wang T, Tian R, Ji W, Yu M, Zhao Y *et al*: **A novel Anxa2-interacting protein Ebp1 inhibits cancer proliferation and invasion by suppressing Anxa2 protein level**. *Mol Cell Endocrinol* 2015, **411**:75-85.

83. Linge A, Maurya P, Friedrich K, Baretton GB, Kelly S, Henry M, Clynes M, Larkin A, Meleady P: **Identification and functional validation of RAD23B as a potential protein in human breast cancer progression**. *J Proteome Res* 2014, **13**(7):3212-3222.

84. Yasui K, Arii S, Zhao C, Imoto I, Ueda M, Nagai H, Emi M, Inazawa J: **TFDP1, CUL4A, and CDC16 identified as targets for amplification at 13q34 in hepatocellular carcinomas**. *Hepatology* 2002, **35**(6):1476-1484.

85. Wang Y, Wen M, Kwon Y, Xu Y, Liu Y, Zhang P, He X, Wang Q, Huang Y, Jen KY *et al*: **CUL4A induces epithelial-mesenchymal transition and promotes cancer metastasis by regulating ZEB1 expression**. *Cancer Res* 2014, **74**(2):520-531.

86. McGee AM, Douglas DL, Liang Y, Hyder SM, Baines CP: **The mitochondrial protein C1qbp promotes cell proliferation, migration and resistance to cell death**. *Cell Cycle* 2011, **10**(23):4119-4127.

87. Saha P, Ghosh I, Datta K: **Increased hyaluronan levels in HABP1/p32/gC1qR overexpressing HepG2 cells inhibit autophagic vacuolation regulating tumor potency**. *PLoS One* 2014, **9**(7):e103208.

88. Tucker LA, Zhang Q, Sheppard GS, Lou P, Jiang F, McKeegan E, Lesniewski R, Davidsen SK, Bell RL, Wang J: **Ectopic expression of methionine aminopeptidase-2 causes cell transformation and stimulates proliferation**. *Oncogene* 2008, **27**(28):3967-3976.

89. Warder SE, Tucker LA, McLoughlin SM, Strelitzer TJ, Meuth JL, Zhang Q, Sheppard GS, Richardson PL, Lesniewski R, Davidsen SK *et al*: **Discovery, Identification, and Characterization of Candidate Pharmacodynamic Markers of Methionine Aminopeptidase-2 Inhibition**. *Journal of Proteome Research* 2008, **7**(11):4807-4820.

90. Shimizu H, Yamagishi S, Chiba H, Ghazizadeh M: **Methionine Aminopeptidase 2 as a Potential Therapeutic Target for Human Non-Small-Cell Lung Cancers**. *Adv Clin Exp Med* 2016, **25**(1):117-128.

91. Kim SS, Hur SY, Kim YR, Yoo NJ, Lee SH: **Expression of AIMP1, 2 and 3, the scaffolds for the multi-tRNA synthetase complex, is downregulated in gastric and colorectal cancer**. *Tumori* 2011, **97**(3):380-385.

92. Butt AJ, Sergio CM, Inman CK, Anderson LR, McNeil CM, Russell AJ, Nousch M, Preiss T, Biankin AV, Sutherland RL *et al*: **The estrogen and c-Myc target gene HSPC111 is over-expressed in breast cancer and associated with poor patient outcome**. *Breast Cancer Res* 2008, **10**(2):R28.

93. He H-c, Ling X-h, Zhu J-g, Fu X, Han Z-d, Liang Y-x, Deng Y-h, Lin Z-y, Chen G, Chen Y-f *et al*: **Down-regulation of the ErbB3 binding protein 1 in human bladder cancer promotes tumor progression and cell proliferation**. *Molecular Biology Reports* 2013, **40**(5):3799-3805.

94. Liu L, Li XD, Chen HY, Cui JS, Xu DY: **Significance of Ebp1 and p53 protein expression in cervical cancer**. *Genet Mol Res* 2015, **14**(4):11860-11866.

95. Tamaki M, Goi T, Hirono Y, Katayama K, Yamaguchi A: **PPP2R1B gene alterations inhibit interaction of PP2A-Abeta and PP2A-C proteins in colorectal cancers**. *Oncol Rep* 2004, **11**(3):655-659.

96. Zhang Y, Talmon G, Wang J: **MicroRNA-587 antagonizes 5-FU-induced apoptosis and confers drug resistance by regulating PPP2R1B expression in colorectal cancer**. *Cell Death Dis* 2015, **6**(8):e1845.

97. Avram S, Mernea M, Mihailescu DF, Seiman CD, Seiman DD, Putz MV: **Mitotic checkpoint proteins Mad1 and Mad2 - structural and functional relationship with implication in genetic diseases**. *Curr Comput Aided Drug Des* 2014, **10**(2):168-181.

98. Du J, Du Q, Zhang Y, Sajdik C, Ruan Y, Tian XX, Fang WG: **Expression of cell-cycle regulatory proteins BUBR1, MAD2, Aurora A, cyclin A and cyclin E in invasive ductal breast carcinomas**. *Histol Histopathol* 2011, **26**(6):761-768.

99. Choi JW, Kim Y, Lee JH, Kim YS: **High expression of spindle assembly checkpoint proteins CDC20 and MAD2 is associated with poor prognosis in urothelial bladder cancer**. *Virchows Arch* 2013, **463**(5):681-687.

100. Ledoux AC, Sellier H, Gillies K, Iannetti A, James J, Perkins ND: **NFκB regulates expression of Polo-like kinase 4**. *Cell Cycle* 2013, **12**(18):3052-3062.

101. Sampson PB, Liu Y, Patel NK, Feher M, Forrest B, Li SW, Edwards L, Laufer R, Lang Y, Ban F *et al*: **The discovery of Polo-like kinase 4 inhibitors: design and optimization of spiro[cyclopropane-1,3'[3H]indol]-2'(1'H).ones as orally bioavailable antitumor agents**. *J Med Chem* 2015, **58**(1):130-146.

102. Wenqi D, Li W, Shanshan C, Bei C, Yafei Z, Feihu B, Jie L, Daiming F: **EpCAM is overexpressed in gastric cancer and its downregulation suppresses proliferation of gastric cancer**. *Journal of Cancer Research and Clinical Oncology* 2009, **135**(9):1277-1285.

103. Wang G, Zhang Z, Ren Y: **TROP-1/Ep-CAM and CD24 are potential candidates for ovarian cancer therapy**. *Int J Clin Exp Pathol* 2015, **8**(5):4705-4714.

104. Gao J, Yan Q, Liu S, Yang X: **Knockdown of EpCAM enhances the chemosensitivity of breast cancer cells to 5-fluorouracil by downregulating the antiapoptotic factor Bcl-2**. *PLoS One* 2014, **9**(7):e102590.

105. Santin AD, Cane S, Bellone S, Bignotti E, Palmieri M, De Las Casas LE, Anfossi S, Roman JJ, O'Brien T, Pecorelli S: **The novel serine protease tumor-associated differentially expressed gene-15 (matriptase/MT-SP1) is highly overexpressed in cervical carcinoma**. *Cancer* 2003, **98**(9):1898-1904.

106. Singh PK, Srivastava AK, Dalela D, Rath SK, Goel MM, Bhatt ML: **Frequent expression of zinc-finger protein ZNF165 in human urinary bladder transitional cell carcinoma**. *Immunobiology* 2015, **220**(1):68-73.

107. Dong W, Chen X, Xie J, Sun P, Wu Y: **Epigenetic inactivation and tumor suppressor activity of HAI-2/SPINT2 in gastric cancer**. *Int J Cancer* 2010, **127**(7):1526-1534.

108. Suzuki M, Kobayashi H, Tanaka Y, Hirashima Y, Kanayama N, Takei Y, Saga Y, Suzuki M, Itoh H, Terao T: **Suppression of invasion and peritoneal carcinomatosis of ovarian cancer cell line by overexpression of bikunin**. *Int J Cancer* 2003, **104**(3):289-302.

109. Ikeo K, Oshima T, Shan J, Matsui H, Tomita T, Fukui H, Watari J, Miwa H: **Junctional adhesion molecule-A promotes proliferation and inhibits apoptosis of gastric cancer**. *Hepatogastroenterology* 2015, **62**(138):540-545.

110. Tian Y, Tian Y, Zhang W, Wei F, Yang J, Luo X, Zhou T, Hou B, Qian S, Deng X *et al*: **Junctional adhesion molecule-A, an epithelial-mesenchymal transition inducer, correlates with metastasis and poor prognosis in human nasopharyngeal cancer**. *Carcinogenesis* 2015, **36**(1):41-48.

111. Yang X, Cao W, Zhou J, Zhang W, Zhang X, Lin W, Fei Z, Lin H, Wang B: **14-3-3ζ positive expression is associated with a poor prognosis in patients with glioblastoma**. *Neurosurgery* 2011, **68**(4):932-938; discussion 938.

112. Rüenauver K, Menon R, Svensson MA, Carlsson J, Vogel W, Andrén O, Nowak M, Perner S: **Prognostic significance of YWHAZ expression in localized prostate cancer**. *Prostate Cancer Prostatic Dis* 2014, **17**(4):310-314.

113. Bergamaschi A, Frasor J, Borgen K, Stanculescu A, Johnson P, Rowland K, Wiley EL, Katzenellenbogen BS: **14-3-3ζ as a predictor of early time to recurrence and distant metastasis in hormone receptor-positive and -negative breast cancers**. *Breast Cancer Res Treat* 2013, **137**(3):689-696.

114. Cuevas R, Korzeniewski N, Tolstov Y, Hohenfellner M, Duensing S: **FGF-2 disrupts mitotic stability in prostate cancer through the intracellular trafficking protein CEP57**. *Cancer Res* 2013, **73**(4):1400-1410.

115. Bilke S, Schwentner R, Yang F, Kauer M, Jug G, Walker RL, Davis S, Zhu YJ, Pineda M, Meltzer PS *et al*: **Oncogenic ETS fusions deregulate E2F3 target genes in Ewing sarcoma and prostate cancer**. *Genome Res* 2013, **23**(11):1797-1809.

116. Giorgi C, Boro A, Rechfeld F, Lopez-Garcia LA, Gierisch ME, Schäfer BW, Niggli FK: **PI3K/AKT signaling modulates transcriptional expression of EWS/FLI1 through specificity protein 1**. *Oncotarget* 2015, **6**(30):28895-28910.

117. Tsofack SP, Meunier L, Sanchez L, Madore J, Provencher D, Mes-Masson AM, Lebel M: **Low expression of the X-linked ribosomal protein S4 in human serous epithelial ovarian cancer is associated with a poor prognosis**. *BMC Cancer* 2013, **13**:303.

118. Paquet É R, Hovington H, Brisson H, Lacombe C, Larue H, Têtu B, Lacombe L, Fradet Y, Lebel M: **Low level of the X-linked ribosomal protein S4 in human urothelial carcinomas is associated with a poor prognosis**. *Biomark Med* 2015, **9**(3):187-197.

119. Zhou X, Liu Y, You J, Zhang H, Zhang X, Ye L: **Myosin light-chain kinase contributes to the proliferation and migration of breast cancer cells through cross-talk with activated ERK1/2**. *Cancer Lett* 2008, **270**(2):312-327.

120. Chen L, Su L, Li J, Zheng Y, Yu B, Yu Y, Yan M, Gu Q, Zhu Z, Liu B: **Hypermethylated FAM5C and MYLK in serum as diagnosis and pre-warning markers for gastric cancer**. *Dis Markers* 2012, **32**(3):195-202.

121. Ducker CE, Upson JJ, French KJ, Smith CD: **Two N-myristoyltransferase isozymes play unique roles in protein myristoylation, proliferation, and apoptosis**. *Mol Cancer Res* 2005, **3**(8):463-476.

122. Luo H, Hao X, Ge C, Zhao F, Zhu M, Chen T, Yao M, He X, Li J: **TC21 promotes cell motility and metastasis by regulating the expression of E-cadherin and N-cadherin in hepatocellular carcinoma**. *Int J Oncol* 2010, **37**(4):853-859.

123. Gutierrez-Erlandsson S, Herrero-Vidal P, Fernandez-Alfara M, Hernandez-Garcia S, Gonzalo-Flores S, Mudarra-Rubio A, Fresno M, Cubelos B: **R-RAS2 overexpression in tumors of the human central nervous system**. *Mol Cancer* 2013, **12**(1):127.

124. Larive RM, Moriggi G, Menacho-Márquez M, Cañamero M, de Álava E, Alarcón B, Dosil M, Bustelo XR: **Contribution of the R-Ras2 GTP-binding protein to primary breast tumorigenesis and late-stage metastatic disease**. *Nat Commun* 2014, **5**:3881.

125. Zhou B, Wu Q, Chen G, Zhang TP, Zhao YP: **NOP14 promotes proliferation and metastasis of pancreatic cancer cells**. *Cancer Lett* 2012, **322**(2):195-203.

126. Sheng X, Bowen N, Wang Z: **GLI pathogenesis-related 1 functions as a tumor-suppressor in lung cancer**. *Mol Cancer* 2016, **15**:25.

127. Li L, Ren C, Yang G, Fattah EA, Goltsov AA, Kim SM, Lee JS, Park S, Demayo FJ, Ittmann MM *et al*: **GLIPR1 suppresses prostate cancer development through targeted oncoprotein destruction**. *Cancer Res* 2011, **71**(24):7694-7704.

128. Shi L, Zhang B, Sun X, Lu S, Liu Z, Liu Y, Li H, Wang L, Wang X, Zhao C: **MiR-204 inhibits human NSCLC metastasis through suppression of NUAK1**. *Br J Cancer* 2014, **111**(12):2316-2327.

129. Bell RE, Khaled M, Netanely D, Schubert S, Golan T, Buxbaum A, Janas MM, Postolsky B, Goldberg MS, Shamir R *et al*: **Transcription factor/microRNA axis blocks melanoma invasion program by miR-211 targeting NUAK1**. *J Invest Dermatol* 2014, **134**(2):441-451.

130. Hou X, Liu JE, Liu W, Liu CY, Liu ZY, Sun ZY: **A new role of NUAK1: directly phosphorylating p53 and regulating cell proliferation**. *Oncogene* 2011, **30**(26):2933-2942.

131. Lee SY, Kim JW, Jeong MH, An JH, Jang SM, Song KH, Choi KH: **Microtubule-associated protein 1B light chain (MAP1B-LC1) negatively regulates the activity of tumor suppressor p53 in neuroblastoma cells**. *FEBS Lett* 2008, **582**(19):2826-2832.

132. Guo MM, Hu LH, Wang YQ, Chen P, Huang JG, Lu N, He JH, Liao CG: **miR-22 is down-regulated in gastric cancer, and its overexpression inhibits cell migration and invasion via targeting transcription factor Sp1**. *Med Oncol* 2013, **30**(2):542.

133. Zhang G, Xia S, Tian H, Liu Z, Zhou T: **Clinical significance of miR-22 expression in patients with colorectal cancer**. *Med Oncol* 2012, **29**(5):3108-3112.

134. Wang J, Sun D, Wang Y, Ren F, Pang S, Wang D, Xu S: **FOSL2 positively regulates TGF-β1 signalling in non-small cell lung cancer**. *PLoS One* 2014, **9**(11):e112150.

135. Milde-Langosch K, Janke S, Wagner I, Schröder C, Streichert T, Bamberger AM, Jänicke F, Löning T: **Role of Fra-2 in breast cancer: influence on tumor cell invasion and motility**. *Breast Cancer Res Treat* 2008, **107**(3):337-347.

136. Malaguti C, Rossini GP: **Recovery of cellular E-cadherin precedes replenishment of estrogen receptor and estrogen-dependent proliferation of breast cancer cells rescued from a death stimulus**. *J Cell Physiol* 2002, **192**(2):171-181.

137. Li YJ, Ji XR: **Relationship between expression of E-cadherin-catenin complex and clinicopathologic characteristics of pancreatic cancer**. *World J Gastroenterol* 2003, **9**(2):368-372.

138. Rodriguez C, Borgel J, Court F, Cathala G, Forné T, Piette J: **CTCF is a DNA methylation-sensitive positive regulator of the INK/ARF locus**. *Biochem Biophys Res Commun* 2010, **392**(2):129-134.

139. Docquier F, Farrar D, Arcy V, Chernukhin I, Robinson AF, Loukinov D, Vatolin S, Pack S, Mackay A, Harris RA *et al*: **Heightened Expression of CTCF in Breast Cancer Cells Is Associated with Resistance to Apoptosis**. *Cancer Research* 2005, **65**(12):5112.

140. Tane S, Sakai Y, Hokka D, Okuma H, Ogawa H, Tanaka Y, Uchino K, Nishio W, Yoshimura M, Maniwa Y: **Significant role of Psf3 expression in non-small-cell lung cancer**. *Cancer Sci* 2015, **106**(11):1625-1634.

141. Hattori M, Minato N: **Rap1 GTPase: functions, regulation, and malignancy**. *J Biochem* 2003, **134**(4):479-484.

142. Tsygankova OM, Wang H, Meinkoth JL: **Tumor cell migration and invasion are enhanced by depletion of Rap1 GTPase-activating protein (Rap1GAP)**. *J Biol Chem* 2013, **288**(34):24636-24646.

143. Tsygankova OM, Feshchenko E, Klein PS, Meinkoth JL: **Thyroid-stimulating hormone/cAMP and glycogen synthase kinase 3beta elicit opposing effects on Rap1GAP stability**. *J Biol Chem* 2004, **279**(7):5501-5507.

144. Srivastava A, Alexander J, Lomakin I, Dayal Y: **Immunohistochemical expression of transforming growth factor alpha and epidermal growth factor receptor in pancreatic endocrine tumors**. *Hum Pathol* 2001, **32**(11):1184-1189.

145. Rhee J, Han SW, Cha Y, Ham HS, Kim HP, Oh DY, Im SA, Park JW, Ro J, Lee KS *et al*: **High serum TGF-α predicts poor response to lapatinib and capecitabine in HER2-positive breast cancer**. *Breast Cancer Res Treat* 2011, **125**(1):107-114.

146. Wasniewski T, Woclawek-Potocka I, Boruszewska D, Kowalczyk-Zieba I, Sinderewicz E, Grycmacher K: **The significance of the altered expression of lysophosphatidic acid receptors, autotaxin and phospholipase A2 as the potential biomarkers in type 1 endometrial cancer biology**. *Oncol Rep* 2015, **34**(5):2760-2767.

147. Fujita T, Miyamoto S, Onoyama I, Sonoda K, Mekada E, Nakano H: **Expression of lysophosphatidic acid receptors and vascular endothelial growth factor mediating lysophosphatidic acid in the development of human ovarian cancer**. *Cancer Lett* 2003, **192**(2):161-169.

148. Lai YH, He RY, Chou JL, Chan MW, Li YF, Tai CK: **Promoter hypermethylation and silencing of tissue factor pathway inhibitor-2 in oral squamous cell carcinoma**. *J Transl Med* 2014, **12**:237.

149. Makishima H, Visconte V, Sakaguchi H, Jankowska AM, Abu Kar S, Jerez A, Przychodzen B, Bupathi M, Guinta K, Afable MG *et al*: **Mutations in the spliceosome machinery, a novel and ubiquitous pathway in leukemogenesis**. *Blood* 2012, **119**(14):3203-3210.

150. Edmond V, Merdzhanova G, Gout S, Brambilla E, Gazzeri S, Eymin B: **A new function of the splicing factor SRSF2 in the control of E2F1-mediated cell cycle progression in neuroendocrine lung tumors**. *Cell Cycle* 2013, **12**(8):1267-1278.

151. Nemoto K, Vogt A, Oguri T, Lazo JS: **Activation of the Raf-1/MEK/Erk kinase pathway by a novel Cdc25 inhibitor in human prostate cancer cells**. *Prostate* 2004, **58**(1):95-102.

152. Mi Y, Thomas SD, Xu X, Casson LK, Miller DM, Bates PJ: **Apoptosis in leukemia cells is accompanied by alterations in the levels and localization of nucleolin**. *J Biol Chem* 2003, **278**(10):8572-8579.

153. Zhang R, Zhang Y, Li H: **miR-1244/Myocyte Enhancer Factor 2D Regulatory Loop Contributes to the Growth of Lung Carcinoma**. *DNA Cell Biol* 2015, **34**(11):692-700.

154. Li J, Tan M, Li L, Pamarthy D, Lawrence TS, Sun Y: **SAK, a new polo-like kinase, is transcriptionally repressed by p53 and induces apoptosis upon RNAi silencing**. *Neoplasia* 2005, **7**(4):312-323.

155. Fan G, Sun L, Shan P, Zhang X, Huan J, Zhang X, Li D, Wang T, Wei T, Zhang X *et al*: **Loss of KLF14 triggers centrosome amplification and tumorigenesis**. *Nat Commun* 2015, **6**:8450.

156. Peng ZG, Yao YB, Yang J, Tang YL, Huang X: **Mangiferin induces cell cycle arrest at G2/M phase through ATR-Chk1 pathway in HL-60 leukemia cells**. *Genet Mol Res* 2015, **14**(2):4989-5002.

157. Cho SH, Toouli CD, Fujii GH, Crain C, Parry D: **Chk1 is essential for tumor cell viability following activation of the replication checkpoint**. *Cell Cycle* 2005, **4**(1):131-139.

158. Varga AE, Stourman NV, Zheng Q, Safina AF, Quan L, Li X, Sossey-Alaoui K, Bakin AV: **Silencing of the Tropomyosin-1 gene by DNA methylation alters tumor suppressor function of TGF-beta**. *Oncogene* 2005, **24**(32):5043-5052.

159. Wang J, Guan J, Lu Z, Jin J, Cai Y, Wang C, Wang F: **Clinical and tumor significance of tropomyosin-1 expression levels in renal cell carcinoma**. *Oncol Rep* 2015, **33**(3):1326-1334.

160. Kristiansen G, Denkert C, Schlüns K, Dahl E, Pilarsky C, Hauptmann S: **CD24 is expressed in ovarian cancer and is a new independent prognostic marker of patient survival**. *Am J Pathol* 2002, **161**(4):1215-1221.

161. Zhu J, Nie S, Wu J, Lubman DM: **Target proteomic profiling of frozen pancreatic CD24+ adenocarcinoma tissues by immuno-laser capture microdissection and nano-LC-MS/MS**. *J Proteome Res* 2013, **12**(6):2791-2804.

162. Gajulapalli VNarasihma R, Samanthapudi VSubramanyam K, Pulaganti M, Khumukcham Saratchandra S, Malisetty Vijaya L, Guruprasad L, Chitta Suresh K, Manavathi B: **A transcriptional repressive role for epithelial-specific ETS factor ELF3 on oestrogen receptor alpha in breast cancer cells**. *Biochemical Journal* 2016, **473**(8):1047-1061.

163. Kohno Y, Okamoto T, Ishibe T, Nagayama S, Shima Y, Nishijo K, Shibata KR, Fukiage K, Otsuka S, Uejima D *et al*: **Expression of claudin7 is tightly associated with epithelial structures in synovial sarcomas and regulated by an Ets family transcription factor, ELF3**. *J Biol Chem* 2006, **281**(50):38941-38950.

164. Man X, He J, Kong C, Zhu Y, Zhang Z: **Clinical significance and biological roles of CARMA3 in human bladder carcinoma**. *Tumour Biol* 2014, **35**(5):4131-4136.

165. Xia ZX, Li ZX, Zhang M, Sun LM, Zhang QF, Qiu XS: **CARMA3 regulates the invasion, migration, and apoptosis of non-small cell lung cancer cells by activating NF-кB and suppressing the P38 MAPK signaling pathway**. *Exp Mol Pathol* 2016, **100**(2):353-360.

166. Mehdipour P, Pirouzpanah S, Sarafnejad A, Atri M, Shahrestani TS, Haidari M: **Prognostic implication of CDC25A and cyclin E expression on primary breast cancer patients**. *Cell Biology International* 2009, **33**(10):1050-1056.

167. Adler AS, McCleland ML, Truong T, Lau S, Modrusan Z, Soukup TM, Roose-Girma M, Blackwood EM, Firestein R: **CDK8 maintains tumor dedifferentiation and embryonic stem cell pluripotency**. *Cancer Res* 2012, **72**(8):2129-2139.

168. Clark AD, Oldenbroek M, Boyer TG: **Mediator kinase module and human tumorigenesis**. *Crit Rev Biochem Mol Biol* 2015, **50**(5):393-426.

169. Mohamed ER, Naito M, Terasaki Y, Niu Y, Gohara S, Komatsu N, Shichijo S, Itoh K, Noguchi M: **Capability of SART3(109-118) peptide to induce cytotoxic T lymphocytes from prostate cancer patients with HLA class I-A11, -A31 and -A33 alleles**. *Int J Oncol* 2009, **34**(2):529-536.

170. Sasatomi T, Suefuji Y, Matsunaga K, Yamana H, Miyagi Y, Araki Y, Ogata Y, Itoh K, Shirouzu K: **Expression of tumor rejection antigens in colorectal carcinomas**. *Cancer* 2002, **94**(6):1636-1641.

171. Landrette SF, Kuo YH, Hensen K, Barjesteh van Waalwijk van Doorn-Khosrovani S, Perrat PN, Van de Ven WJ, Delwel R, Castilla LH: **Plag1 and Plagl2 are oncogenes that induce acute myeloid leukemia in cooperation with Cbfb-MYH11**. *Blood* 2005, **105**(7):2900-2907.

172. Hanks TS, Gauss KA: **Pleomorphic adenoma gene-like 2 regulates expression of the p53 family member, p73, and induces cell cycle block and apoptosis in human promonocytic U937 cells**. *Apoptosis* 2012, **17**(3):236-247.

173. Liu B, Lu C, Song YX, Gao P, Sun JX, Chen XW, Wang MX, Dong YL, Xu HM, Wang ZN: **The role of pleomorphic adenoma gene-like 2 in gastrointestinal cancer development, progression, and prognosis**. *Int J Clin Exp Pathol* 2014, **7**(6):3089-3100.

174. Wang JL, Chen ZF, Chen HM, Wang MY, Kong X, Wang YC, Sun TT, Hong J, Zou W, Xu J *et al*: **Elf3 drives β-catenin transactivation and associates with poor prognosis in colorectal cancer**. *Cell Death Dis* 2014, **5**(5):e1263-e1263.

175. Liu C, Zhang L, Huang Y, Lu K, Tao T, Chen S, Zhang X, Guan H, Chen M, Xu B: **MicroRNA‑328 directly targets p21‑activated protein kinase 6 inhibiting prostate cancer proliferation and enhancing docetaxel sensitivity**. *Mol Med Rep* 2015, **12**(5):7389-7395.

176. Liu X, Busby J, John C, Wei J, Yuan X, Lu ML: **Direct interaction between AR and PAK6 in androgen-stimulated PAK6 activation**. *PLoS One* 2013, **8**(10):e77367.

177. Blum C, Graham A, Yousefzadeh M, Shrout J, Benjamin K, Krishna M, Hoda R, Hoda R, Cole DJ, Garrett-Mayer E *et al*: **The expression ratio of Map7/B2M is prognostic for survival in patients with stage II colon cancer**. *Int J Oncol* 2008, **33**(3):579-584.

178. Biliran H, Jr., Sheng S: **Pleiotrophic inhibition of pericellular urokinase-type plasminogen activator system by endogenous tumor suppressive maspin**. *Cancer Res* 2001, **61**(24):8676-8682.

179. Ashida S, Furihata M, Katagiri T, Tamura K, Anazawa Y, Yoshioka H, Miki T, Fujioka T, Shuin T, Nakamura Y *et al*: **Expression of novel molecules, MICAL2-PV (MICAL2 prostate cancer variants), increases with high Gleason score and prostate cancer progression**. *Clin Cancer Res* 2006, **12**(9):2767-2773.

180. Dallas SL, Zhao S, Cramer SD, Chen Z, Peehl DM, Bonewald LF: **Preferential production of latent transforming growth factor beta-2 by primary prostatic epithelial cells and its activation by prostate-specific antigen**. *J Cell Physiol* 2005, **202**(2):361-370.

181. Wick W, Platten M, Weller M: **Glioma cell invasion: regulation of metalloproteinase activity by TGF-beta**. *J Neurooncol* 2001, **53**(2):177-185.

182. Ohno Y, Izumi M, Kawamura T, Nishimura T, Mukai K, Tachibana M: **Annexin II represents metastatic potential in clear-cell renal cell carcinoma**. *Br J Cancer* 2009, **101**(2):287-294.

183. Stephenson JM, Banerjee S, Saxena NK, Cherian R, Banerjee SK: **Neuropilin-1 is differentially expressed in myoepithelial cells and vascular smooth muscle cells in preneoplastic and neoplastic human breast: a possible marker for the progression of breast cancer**. *Int J Cancer* 2002, **101**(5):409-414.
